# Liquid-activated quantum emission from pristine hexagonal boron nitride for nanofluidic sensing

**DOI:** 10.1038/s41563-023-01658-2

**Published:** 2023-08-31

**Authors:** Nathan Ronceray, Yi You, Evgenii Glushkov, Martina Lihter, Benjamin Rehl, Tzu-Heng Chen, Gwang-Hyeon Nam, Fanny Borza, Kenji Watanabe, Takashi Taniguchi, Sylvie Roke, Ashok Keerthi, Jean Comtet, Boya Radha, Aleksandra Radenovic

**Affiliations:** 1 Laboratory of Nanoscale Biology, Institute of Bioengineering (IBI), School of Engineering (STI), École Polytechnique Fédérale de Lausanne (EPFL), Lausanne, Switzerland; 2 Laboratory for Fundamental BioPhotonics, Institute of Bioengineering (IBI), School of Engineering (STI), École Polytechnique Fédérale de Lausanne, Lausanne, Switzerland; 3 Department of Physics and Astronomy, School of Natural Sciences, The University of Manchester, Manchester, UK; 4 National Graphene Institute, The University of Manchester, Manchester, UK; 5 Research Center for Electronic and Optical Materials, National Institute for Materials Science, Tsukuba, Japan; 6 Research Center for Materials Nanoarchitectonics, National Institute for Materials Science, Tsukuba, Japan; 7 Department of Chemistry, School of Natural Sciences, The University of Manchester, Manchester, UK; 8 Soft Matter Sciences and Engineering, ESPCI Paris, PSL University, CNRS, Sorbonne Université, Paris, France; 9 Present Address: Institute of Physics, Zagreb, Croatia

**Keywords:** Two-dimensional materials, Nanofluidics

## Abstract

Liquids confined down to the atomic scale can show radically new properties. However, only indirect and ensemble measurements operate in such extreme confinement, calling for novel optical approaches that enable direct imaging at the molecular level. Here we harness fluorescence originating from single-photon emitters at the surface of hexagonal boron nitride for molecular imaging and sensing in nanometrically confined liquids. The emission originates from the chemisorption of organic solvent molecules onto native surface defects, revealing single-molecule dynamics at the interface through the spatially correlated activation of neighbouring defects. Emitter spectra further offer a direct readout of the local dielectric properties, unveiling increasing dielectric order under nanometre-scale confinement. Liquid-activated native hexagonal boron nitride defects bridge the gap between solid-state nanophotonics and nanofluidics, opening new avenues for nanoscale sensing and optofluidics.

## Abstract (web summary)

Two-dimensional crystals of hexagonal boron nitride become fluorescent when immersed in common solvents. Now, this phenomenon is used in the design of in-liquid sensors operating at the nanometre scale.

## Main

Nanostructures made of two-dimensional (2D) materials have become prominent in nanofluidic research^1,2^. Liquid confinement to a few molecular layers between atomically smooth walls has led to anomalies in molecular transport^3–6^ and structure^7,8^. Nevertheless, a direct observation of these emerging phenomena remains challenging due to the limitations of current techniques in extreme confinements, where even molecular fluorophores cannot penetrate^9^. This calls for the development of imaging methods that can access molecular properties in confinement^1,2^.

To tackle this goal, solid-state optically active defects show promise. Fluorescent defects in diamond have enabled the optical probing of nanoscale matter^10^, including liquids^11^, but they cannot be easily embedded in 2D nanostructures. Coincidentally, hexagonal boron nitride (hBN) has been used in both nanofluidics^6,8,12^ and nanophotonics, where various point defects within its 6 eV bandgap have been identified as room-temperature quantum emitters^13–15^. Although emitters in hBN have been artificially induced using techniques such as irradiation^16,17^ or carbon doping^18,19^, the potential of liquid treatments remains largely unexplored. Recent studies have combined liquid and irradiation treatments to activate plasma-induced surface defects in hBN using water^20^ and binary mixtures of water with organic solvents^21^. Post-treatment of ion-beam-exposed hBN with liquids has also been shown to modify the defect emission properties^22^. Furthermore, plasma-induced surface defects have been utilized to study single interfacial charge dynamics, such as proton hopping^20,21^. However, even mild plasma treatment induces mechanical and chemical changes that result in hBN crystals no longer having atomically smooth surfaces^23^, thus preventing the integration of defects in ultraflat van der Waals heterostructures, which are crucial for advancements in ångström-scale fluidics^4,8^.

In this study, we demonstrate that organic solvents can activate visible-range quantum emission from pristine high-quality hBN crystals. We attribute this phenomenon to the interaction between organic molecules and native surface defects^24–27^. By employing spectral super-resolution microscopy^28^, we observe defect-mediated molecular random walks and couplings between defect dipoles and the liquid medium, leading to tunable emission wavelengths through the dielectric properties of the liquid. Leveraging the intrinsic properties of widely used 2D materials and common solvents, the fluorescence activation mechanism reported here is utilized to image nanofluidic structures, with emitters serving as the nanoscale probes of the order and dynamics of liquid media confined to the nanoscale.

### Liquid-activated fluorescence from pristine hBN

**Fig. 1:**
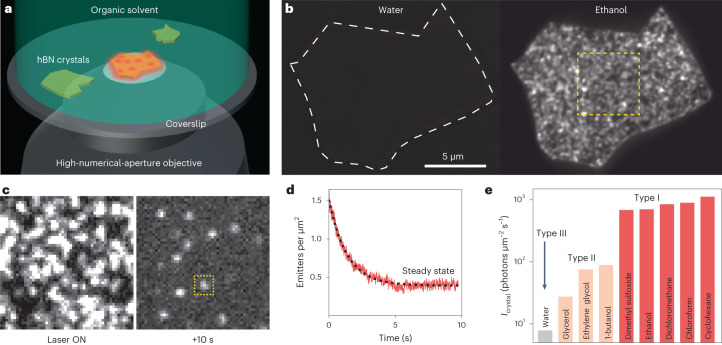
Liquid-induced fluorescence from pristine hBN crystals. **a**, Sketch of the experimental setup. **b**, Wide-field fluorescence images of an hBN crystal under 3.5 kW cm^–2^ and 561 nm laser light illumination with 1 s exposure time. No fluorescence was observed in water, but in ethanol, the entire crystal surface became fluorescent. The images underwent linear contrast enhancement. **c**, A zoomed-in view of the dashed yellow box in **b** reveals dense clusters of emission when the laser is turned ON, with 6 ms exposure time. After 10 s of wide-field illumination, the crystal surface reached a stable number of diffraction-limited isolated emitters. **d**, Localization microscopy-based counting of the emitters as a function of illumination time: after 5 s, a steady state was reached. The dashed line is a fit to an offset exponential relaxation. **e**, Liquid dependency of the crystal fluorescence, showing strongly activating liquids (type I), mildly activating liquids (type II) and no activation in water (type III). Source data

The surface of untreated hBN crystals exhibits visible-range fluorescence when in contact with common organic solvents like ethanol. To demonstrate this effect, we exfoliated high-quality hBN crystals^24^ onto a glass coverslip, which was placed in a liquid-filled chamber on an inverted microscope (Fig. 1a). Photoluminescence (PL) of the crystals under 561 nm wide-field laser illumination (0.35–3.50 kW cm^–2^) was collected using a high-numerical-aperture objective and projected onto a camera chip. Pristine hBN in air or water did not exhibit fluorescence under these illumination conditions. However, we observed intense fluorescence from as-exfoliated crystals in contact with ethanol (Fig. 1b). The fluorescence intensity gradually decreased over continuous illumination and stabilized after several seconds, revealing emission from sub-diffraction spots (Fig. 1c and Supplementary Video 1), which can be localized with ~10 nm precision using single-molecule localization microscopy^29^ (Methods). We attribute this emission to the activation of defects present in the as-exfoliated crystal through contact with the liquid, resulting in randomly distributed transient emitters on the surface (Supplementary Fig. 1). Under constant 3.5 kW cm^–2^ illumination, the number of emitters decreased to a stable value of approximately 0.4 per square micron (Fig. 1d). This process does not deteriorate the crystal (Supplementary Figs. 2 and 3), and the decrease observed in Fig. 1c is reversible: when left in the dark, the crystal fluorescence recovered within tens of minutes (Supplementary Fig. 4) without inducing additional emitters in the steady state (Supplementary Fig. 5).

Remarkably, the fluorescent activation of the surface occurred with the most common organic solvents, including *n*-alkanes (pentane to hexadecane) and primary alcohols (methanol to 1-pentanol). However, we did not observe any emission in pure water, heavy water and hydrogen peroxide. To quantitatively compare the steady-state fluorescence in different liquid media, we imaged freshly cleaved hBN crystals in several liquids under 3.5 kW cm^–2^ illumination. The steady-state fluorescence can be quantified using crystal brightness *I*_crystal_, defined as the sum of the localized emitter intensities per surface unit and time unit (Fig. 1e). From these observations, we can classify solvents into three types based on the extent of hBN fluorescent activation. Most organic solvents, such as primary alcohols, *n*-alkanes and chloroalkanes, exhibited intense fluorescence (*I*_crystal_ > 500 photons μm^–2^ s^–1^), representing type-I activation. Glycerol and other high-boiling-point liquids (≥200 °C) exhibited a limited but measurable level of fluorescence, classified as type II. On the other hand, pure water showed no activation, falling into type III (*I*_crystal_ < 10 photons μm^–2^ s^–1^). Furthermore, the addition of 10 v/v% of water in ethanol reduced the number of emitters, resulting in a fivefold reduction in the fluorescence signal (Supplementary Fig. 6). The distinction between activation types could not be solely explained by the physical parameters of the solvents (Supplementary Fig. 7), indicating chemical specificity. The steady-state emitter density was found to depend on the liquid medium (Supplementary Figs. 7 and 8), illumination power (Supplementary Fig. 9) and macroscopic flow over the crystal (Supplementary Fig. 10).

**Fig. 2:**
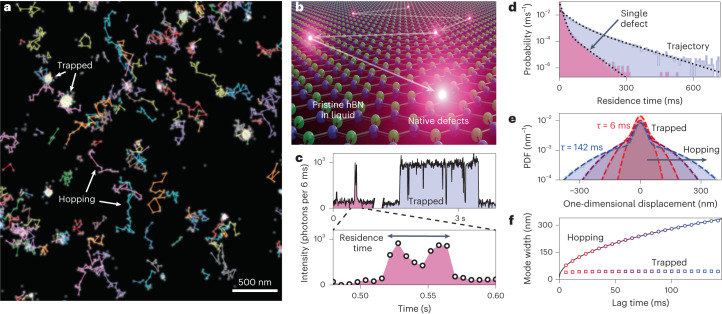
Surface of pristine hBN reveals interfacial molecular dynamics. **a**, Overlay of a super-resolved image from 5,000 frames showing hopping emitters in isopropanol as the linked trajectories, as well as trapped spots. **b**, Artist’s view of the correlated activation of neighbouring defects leading to the trajectories. **c**, Representative intensity traces from the same images, taken from 7 × 7 pixel bins around emitters (dashed yellow box in Fig. 1c) with 6 ms exposure time. The top right trace corresponds to a long defect activation. The top left trace corresponds to a short activation of the same defect, magnified in the bottom panel. **d**, Distribution of residence times on single defects and for the entire trajectories. The dotted lines are fits to a two-component exponential decay. **e**, Displacement probability density functions (PDF) of the trajectories after different lag times *τ* = 6 ms, 24 ms, 66 ms,142 ms. The dashed lines are fits to two-component Gaussians. **f**, Visualizing the evolution of the two modes of the Gaussian fit in **e** with increasing lag time. The central region, corresponding to the trapped state, remains of a constant width, whereas the tails, corresponding to hopping, enlarge with time. The solid line is a fit to a standard diffusion curve. Source data

Considering the dynamics of these fluorescent emitters, a striking observation is the presence of fluorescent trajectories on the crystal surface (Supplementary Video 2). These trajectories indicate the correlated activation of neighbouring defects, corresponding to molecular random walks. By linking the super-resolved localizations of emitters (Methods), we can extract the associated trajectories (Fig. 2b). Previously observed trajectories on plasma-exposed hBN in water and binary mixtures of water and alcohols were attributed to proton hopping^20,21^. A similar phenomenology is thus expected here, with (1) defect activation due to reversible charge transfer from and to the solvent and (2) correlated activation of neighbouring defects, mediated by the lateral motion of charge-bearing solvent molecules that remain physisorbed on the crystal surface. However, the aprotic nature of some solvents used here, as well as the spectral differences, points to a distinct emitter type and reactivity. Since neither pristine hBN nor the liquids used here possess visible-range electronic transitions, the activation of emitters at the interface must arise from a rearrangement of the electronic structure that generates this new optically addressable electronic transition. This could be explained by the chemisorption of organic molecules onto hBN defects^30^. The chemical selectivity (Fig. 1e) suggests that a necessary and sufficient condition for a pure liquid to activate native hBN defects is the presence of a carbon atom in its molecular structure. This finding aligns with recent research, indicating the crucial role of carbon in activating visible-range emitters in hBN^18^. Although a direct observation of the exact chemical structure of the emitters is challenging, the physicochemical interactions between the defects and the liquid along with the photophysical properties of the emitters will guide structural assignment.

### Analysis of emitter dynamics

To quantitatively understand the trajectories, we acquired 50,000 6-ms-long frames of a 13 × 13 μm^2^ crystal area in the steady state, yielding 700,000 localizations leading to 100,000 trajectories. Figure 2a shows a subset of these trajectories overlaid with the super-resolved image obtained from 5,000 frames. This visualization reveals that although some trajectories exhibit a free-hopping behaviour, others remain trapped for extended periods, resulting in bright spots in the super-resolved image. Most emitters are active for tens to hundreds of milliseconds, but a fraction displays stability for several seconds (Fig. 2c), with some activations lasting for over a minute (Supplementary Fig. 11). As measurements were conducted at a low emitter density where trajectories do not split or merge with statistical significance, we assign trajectories to single molecules binding to single defects.

We analysed the observed molecular random walks through their trajectory residence times on the crystal surface ${T}_{\,{{\mbox{res}}}\,}^{{\rm{T}}}$, which comprise complex information on both chemisorption energy at defect sites and physisorption energy on pristine hBN in between defects, as well as the residence times of molecules at single-defect sites ${T}_{\,{{\mbox{res}}}}^{\,{\rm{D}}}$ corresponding to chemisorption only. Both residence times were found to follow a double exponential decay (Fig. 2d), with slow exponential decay components ${\tau }_{\,{{\mbox{res}}}\,}^{{\rm{D}}}$ = 35 ± 1 ms and ${\tau }_{\,{{\mbox{res}}}\,}^{{\rm{T}}}$ = 82 ± 3 ms. Assuming that single-defect residence times follow the Arrhenius equation ${\tau }_{\,{{\mbox{res}}}\,}^{{\rm{D}}}={\nu }^{-1}{{\rm{e}}}^{{{\Delta }}G/kT}$ where *ν* ≈ 10^12^−10^13^ s^−1^ is a molecular attempt rate^21^, we obtain a desorption energy barrier Δ*G* ≈ 24–27*kT* ≈ 0.6–0.7 eV, which is larger than typical physisorption energies (tens of millielectronvolts) and smaller than covalent bonding energies (several electronvolts)^31^. This can be rationalized in terms of a lowered energy barrier under illumination^20^, consistent with the observed light-induced reduction in the number of emitters (Fig. 1d) as well as the illumination power dependency of the density of emitters (Supplementary Fig. 9).

Turning our attention to emitter motion, we find that their one-dimensional displacement probability density function^32^ PDF(*x*,*τ*) follows a two-component Gaussian distribution (Fig. 2e). The central part of the distribution remains of a constant size (~20 nm) corresponding to the localization uncertainty when a trajectory is trapped. The tails of the distribution, however, enlarge with increasing lag time, characterizing the hopping events. As shown in Fig. 2f, the hopping tail size scales as $\sqrt{2D\tau }$ with lag time *τ*, which corresponds to Brownian diffusion with a diffusion coefficient *D* = 9.1 × 10^−14^ m^2^ s^–1^, which is over four orders of magnitude slower than bulk liquid molecular diffusion coefficients. Although this observation makes it impossible to resolve the molecular travel time between defects, this slowdown enables the detection of bright emission from localized spots, which we now propose as a spectral sensing tool.

### Spectral properties and solvatochromic sensing

**Fig. 3:**
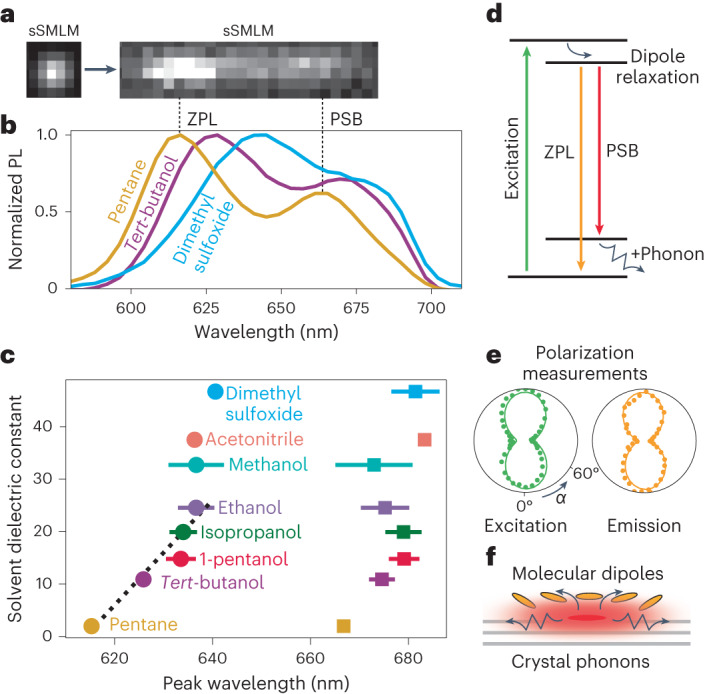
Spectral properties of surface dipole emitters coupled to both solid and liquid environments. **a**, sSMLM splits the fluorescence signal from an emitter into a localization component (left; single-molecule localization microscopy (sSMLM)) and a spectral component (right) on the same camera chip. **b**, Ensemble spectra of liquid-activated emitters in different type-I solvents, exhibiting a clear ZPL and PSB. **c**, Visualizing the wavelength shifts of both ZPL (circles) and PSB (squares), which correlate with the dielectric constant of the liquid. The peak positions were obtained by fitting to a sum of Lorentzians. The dashed line indicates the linear solvatochromic range, with a slope of 1 nm per unit. The error bars correspond to standard deviations of fitting parameters from groups of 100 single-molecule spectra. **d**, Jablonski diagram of processes at play: 561 nm laser excitation induces a dipolar excited state, which can directly emit (orange arrow; ZPL) or with the emission of a phonon (red arrow; PSB). **e**, Normalized intensity as a function of input-light or output-light polarization angle *α* relative to the emitter axis. The solid lines correspond to fits to ideal electric dipole emission cos^2^*α* + constant. Supplementary Fig. 11 provides more details. **f**, Sketch of an excited emitter that can interact with the crystal through phonons as well as with the surrounding molecules (yellow ellipses). Source data

We examined the spectral response of the emitters to their liquid environment using spectral single-molecule localization microscopy (sSMLM)^28^, which enables simultaneous localization and spectral characterization (Fig. 3a and Methods). Single-emitter spectra were found to be homogeneously distributed, with the appearance of a single population of emitters when exposed to the same liquid environment. Ensemble-averaged spectra were consistently characterized by two peaks, classically attributed to the zero-phonon line (ZPL) and the phonon side band (PSB) for emitters embedded in a matrix (Fig. 3b).

Interestingly, these ensemble spectra appeared to strongly depend on the activating liquid, more precisely on its static dielectric constant *ϵ*_liq_. Figure 3b shows the spectra of emitters obtained in the following liquids of increasing polarity: pentane, *tert*-butanol and dimethyl sulfoxide. A notable polarity-induced solvatochromic redshift in the emission was gradually observed from non-polar pentane (615 nm) to the more polar *tert*-butanol (626 nm) to the highly polar dimethyl sulfoxide (641 nm). Beyond the ZPL shift, we observed changes in the PSB, which is less clearly defined for polar solvents. Figure 3c shows the centre wavelengths of both peaks as obtained from fitting to a two-Lorentzian model for several solvents ordered by increasing *ϵ*_liq_. We tested 1-pentanol, isopropanol and methanol on top of previously introduced liquids to interpolate dielectric constant values and found that both ZPL and PSB are redshifted by over 25 nm (~80 meV) in highly polar liquids compared with non-polar alkanes. In the range of *ϵ*_liq_ < 25, a linear dependence was observed between the ZPL wavelength and dielectric constant (Fig. 3c, dashed line), with a slope of approximately 1 nm per unit.

The Jablonski diagram (Fig. 3d) illustrates the process giving rise to the observed spectra; by absorbing a photon (excitation; green arrow), the emitter is excited to a dipolar state, which interacts with the solvent and relaxes before radiating (dipole relaxation; curved arrow). Direct evidence of the dipolar nature of our liquid-activated emitters is presented in Fig. 3e where either the linearly polarized light used for excitation (green) or PL emission (orange) was rotated as the signal was monitored through an analyser (Supplementary Fig. 11). As sketched in Fig. 3f, the solvatochromic redshift can be described by the presence of liquid molecular dipoles stabilizing the excited-state dipole, thus lowering its energy and redshifting the ZPL. After this step (Fig. 3d), the transition back to the ground state can occur in two ways: direct emission of a photon (ZPL; orange arrow) and phonon-assisted emission (PSB; red arrow), which is redshifted compared with the ZPL as a fraction of the energy leads to lattice vibrations (phonon; zigzag arrow). Our analysis of the PSBs revealed increasing phonon broadening and decreasing PSB content with increasing solvent polarity (Supplementary Discussion and Supplementary Fig. 12). In the case of a mixture of polar ethanol and apolar heptane, the spectrum was very similar to that of the polar liquid, demonstrating a strong affinity between the emitters and polar molecules (Supplementary Figs. 13 and 14).

### Time-resolved measurements reveal quantum emission

**Fig. 4:**
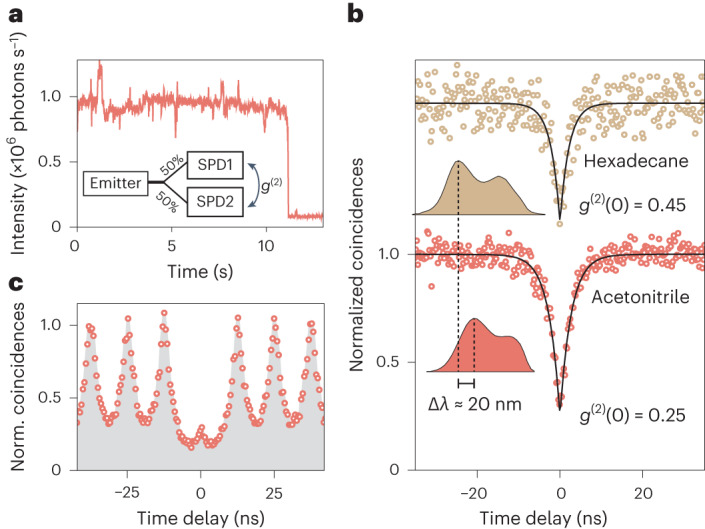
Quantum emission from liquid-activated emitters. **a**, Representative PL trace from an isolated emitter in acetonitrile under 0.7 mW confocal excitation. The shaded region corresponds to the 10-s-long trace used for photon statistics. **b**, Normalized coincidences *g*^(2)^ measured from time-correlated single-photon counting in a Hanbury Brown and Twiss geometry, in hexadecane and acetonitrile. In both liquids, a pronounced antibunching is observed with *g*^(2)^(0) < 0.5 without background correction, proving the single-photon emission. The fluorescent lifetimes, corresponding to the width of the antibunching dip, were found to be 2.73 ± 0.09 ns and 2.20 ± 0.20 ns for acetonitrile and hexadecane, respectively. The spectra are shown in the inset, demonstrating liquid-tunable single-photon emission from 615 to 636 nm. **c**, Single-photon statistics under pulsed laser excitation, showing a suppression of the central peak due to antibunching. Source data

To prove that the measured fluorescence originates from single-photon emitters, we performed time-correlated photon counting. For this, a 0.7 mW continuous-wave 561 nm laser beam was focused to an ~1 μm^2^ spot onto hBN crystals in liquid, and the fluorescence signal was collected by two single-photon detectors in a Hanbury Brown and Twiss interferometer configuration (Fig. 4a, inset). The typical time trace of a stable single emitter in acetonitrile is shown in Fig. 4a. The analysis of the photon arrival times from a 10 s window shows a clear photon antibunching dip *g*^(2)^(0) = 0.25 ± 0.02 at zero delay time, demonstrating single-photon emission (Fig. 4b). This result implies that the bright spots are single emitters and not clusters, and therefore, their optical readout truly reports on the nanoscale properties of the liquid. This activation of quantum emission through the strong chemisorption interaction between a single activating molecule and a single defect was observed in carbon nanotubes^33^, but the mechanism at play here exhibits the particularities of being transient and observed in liquid. Photon statistics under pulsed excitation show the suppression of the correlated pulse peak at zero delay time, confirming single-photon emission (Fig. 4c). This feature was also found in hexadecane with a measured *g*^(2)^(0) = 0.45 ± 0.04. We, thus, demonstrated liquid-tunable single-photon emission with a ZPL shift of 21 nm (Fig. 4b, inset). This shift is comparable with those achieved by hBN defects in response to strain^34^ or electric fields^35^, which shows the potential of liquid-activated emitters as dielectric environment sensors.

### Integration in single-digit nanofluidic systems

**Fig. 5:**
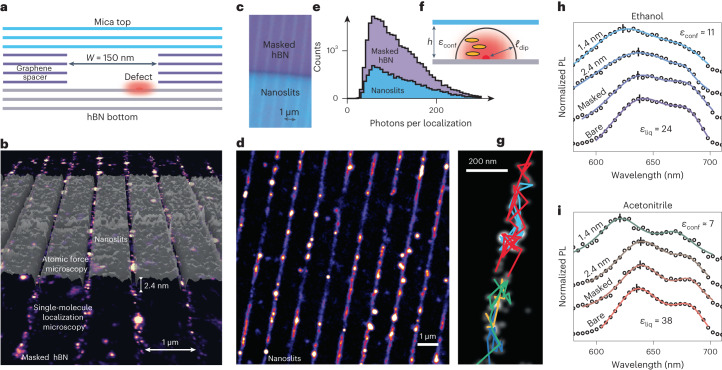
Nanoslit-embedded liquid-activated emitters. **a**, Sketch of the heterostructure nanoslit device. The red glow indicates an emitter inside the nanoslit. **b**, Overlay of a super-resolved image of masked ethanol-activated hBN and the atomic force microscopy mapping of the graphene spacers. **c**, Optical micrograph of the heterostructure. On the purple-coloured part of the image, only graphene spacers on the hBN bottom crystal are present, which leads to masked hBN. The bottom blue region corresponds to the full heterostructure with slits. **d**, Super-resolved image of acetonitrile-activated emitters embedded in 2.4-nm-high nanoslits, from 30,000 frames with 20 ms exposure time and 1.4 kW cm^–2^ illumination. **e**, Comparison of localization intensity distributions for masked hBN and 2.4 nm nanoslits in acetonitrile, showing no loss of photons but an overall reduction in the number of localizations (Supplementary Fig. 18). **f**, Illustration of the effect of confinement: the liquid dielectric constant can be changed by confinement (*ϵ*_conf_) and the defect dipole can interact with solvent molecules (yellow ellipses) within a range *ℓ*_dip_, comparable with the confinement size *h*. **g**, Representative trajectories in the 2.4-nm-high nanoslits filled with ethanol, overlaid with the super-resolved image. **h**,**i**, sSMLM spectra of liquid-activated defects in nanoslits filled with ethanol (**h**) and acetonitrile (**i**). The confinement size is tuned from the open geometry to 2.4 nm down to 1.4 nm. The solid lines correspond to two-component Lorentzian fits, and the black dashes indicate the extracted ZPL position. Source data

Building on the characterization of the emission in bulk liquids, we probed hBN–liquid interfaces in molecular confinement in two-dimensional nanoslits. As sketched in Fig. 5a, the nanoslits are obtained by the van der Waals assembly of heterostructures comprising three crystals: bottom, spacer and top. The top crystal was chosen to be muscovite mica for its transparency and lack of fluorescent properties, and the bottom crystal was pristine hBN to be activated by the liquid. The middle crystal, composed of few-layer graphene patterned by electron-beam lithography, acted as a spacer, defining a slit-shaped channel between the hBN and mica crystals, whose height was set by the number of 3.4-Å-thick graphene layers^4^ (Methods and Supplementary Fig. 15).

An optical micrograph of a device with *h* = 2.4 nm is provided in Fig. 5c. Here the bottom blue region corresponds to the full heterostructure with nanoslits, and the top purple region corresponds to the open hBN crystal masked by graphene spacers but without encapsulation by mica. We verified that covering the pristine hBN crystal with a patterned few-layer graphene crystal masks liquid-activated emitters, as was observed for other types of hBN emitters^36,37^. On bare hBN, emitters are randomly distributed (Supplementary Fig. 16), but the graphene mask allows for their precise positioning on the basal plane of hBN in liquid. An overlay of the graphene-spacer atomic force microscopy topography and the super-resolved image is shown in Fig. 5b, demonstrating the correspondence between the lithographically defined graphene pattern and the optically measured fluorescence from masked hBN. We further verified that capping the masked hBN with mica does not quench its fluorescence, allowing the direct imaging of emitters in a confined liquid (Fig. 5d). Figure 5e shows that the localization intensity distributions with and without the confining mica top are similar, but the number of emitters in confinement are reduced by two-thirds. Single-defect residence times inside nanoslits were found to be slightly longer in nanoslits than in masked hBN (Supplementary Fig. 17). Therefore, the observed threefold decrease in the number of emitters comes not from faster photobleaching of the emitters but from the confinement-induced slowdown of their activation kinetics.

Integrating emitters into nanofluidic structures allows probing the effect of confinement on the liquid structure and dynamics. We first confirm the observation of trajectories in 1.4 nm confinement (Fig. 5g), where a set of emitters is shown in the 150-nm-wide slit. We then focus on the spectral properties of the confined emitters, which can be robustly extracted through sSMLM with relatively low numbers of localizations (<1,000). We present sSMLM spectra obtained in the bare, masked and confined geometries for ethanol (Fig. 5h) and acetonitrile (Fig. 5i). For both solvents, bringing the confinement size from 2.4 nm down to 1.4 nm leads to a clear blueshift (pinpointed by the dashes) indicating the ZPL position. For ethanol, the ZPL blueshifts from 637 to 624 ± 2 nm and from 636 to 621 ± 1 nm for acetonitrile under 1.4 nm confinement, bringing the spectral signature of a strongly polar solvent close to that of non-polar alkanes. This substantial confinement-induced blueshift suggests that emitters experience a reduced dielectric constant of *ϵ*_conf_ ≈ 11 in ethanol and 7 in acetonitrile when considering Fig. 3c as a calibration curve.

Bringing the top mica wall close to the emitters can impact their emission in two ways. First, confinement by the opposite wall of lowered dielectric constant *ϵ*_wall_ ≈ 8 could reduce the effective number of solvent molecules interacting with the emitter and confine the electric field lines within the slit^38^, potentially destabilizing the excited state. Second, the emission can be affected by reducing the out-of-plane component of the liquid dielectric tensor^8^. As depicted in Fig. 5f, the interaction range between a solvent molecule with dipole *μ*_S_ and the defect with dipole *μ*_D_ is given by *ℓ*_dip_ = (*µ*_S_*µ*_D_/2π*ϵ*_o_*k*_B_*T*)^1/3^. Assuming *μ*_D_ ≈ *a* × *e*, where *a* ≈ 0.25 nm is the in-plane lattice parameter of hBN and *e* is the elementary charge, we estimate *μ*_D_ ≈ 12D. Using the dipole moments of ethanol (1.7D) and acetonitrile (3.4D), we find *ℓ*_dip_ ≈ 1.0 and 1.3 nm, respectively, which are smaller than the height of the nanoslit. Hence, the observed effect under 1.4 nm confinement might not solely arise from geometrical effects due to the proximity of the top wall, and could be explained by the confinement-induced reduction in the solvent dielectric constant, as observed for water^8^ and predicted for other liquids^39^. These results consolidate the picture of dipolar-environment-tuned emitters, whose properties are affected by changes in the sensing hemisphere with volume ~2/3π*ℓ*^3^_dip_, which encloses fewer than 100 molecules in the case of acetonitrile. Beyond the passive diffusion and dielectric sensing of confined liquids, tracking emitter dynamics in confinement may be used to directly image the nanoscale flow and study its interplay with defects^40^.

### Outlook

Crystals of hBN, already known for their exceptional optical properties, exhibit a peculiar interaction with liquids. When in contact with organic solvents, native point defects on the atomically smooth surface of the crystal become emissive. This unique system, where the encounter of a single defect with a single organic molecule yields a single-photon emitter, combines solid-state emitters and organic fluorophores, providing a new tool for studying solid–liquid interfaces. We demonstrated two sensing approaches using liquid-activated hBN: the activation dynamics provide insights into interfacial charge transfer between defects and single molecules, whereas the emission spectra of the emitters offer information about the nanoscale dielectric environment. These phenomena were found to hold in confinement as small as a few nanometres, where only ensemble-averaged measurement techniques have been successful so far. As it relies on common samples and widely available single-molecule microscopy techniques, this approach could be readily applied for optical imaging and sensing in nanofluidic systems in operando.

## Methods

### Sample preparation

Pristine hBN flakes from high-quality crystals^24^ were exfoliated onto borosilicate glass coverslips (no. 1.5 Micro Coverglass, Electron Microscopy Sciences, 25 mm in diameter, 170 μm thick), using low-adhesion blue tape. The glass coverslips were cleaned either by (1) sonication in acetone followed by rinsing in isopropanol or (2) sonication in 2% Hellmanex III glassware cleaning agent solution, followed by sonication in deionized water. No differences were observed between (1) and (2) above. The coverslips were rinsed three times in the last solution, after which they were dried with a nitrogen gun covered with a 20-nm-pore filter. Adhesion to the substrate on exfoliation was promoted by oxygen-plasma cleaning of the coverslip (2 min, 100 W). High-quality crystals purchased from HQ Graphene and hBN crystals obtained by the crystallization of hBN from molten iron in a nitrogen–hydrogen atmosphere were also tested, and exhibited no notable difference with the samples grown by us. The crystals were immediately immersed following exfoliation, and the PEEK chamber was thoroughly rinsed three times at each solvent exchange with a fresh solvent. The chamber was covered by an additional glass coverslip to prevent solvent evaporation and contamination. Supplementary Methods gives the fabrication details for the microfluidic flow cell used for flow measurements. Supplementary Table 1 lists the solvents used.

Nanoslits are made of a van der Waals heterostructure of a spacer layer sandwiched between a top layer and a bottom layer following the same protocol as previously reported^4^. Here the van der Waals stack is composed of top mica/graphene spacer/bottom hBN. In brief, thin (few atomic layers) graphene was first patterned via electron-beam lithography into parallel strips with a width of 1 μm and a separation of 150 nm. A mica crystal (~200 nm thick) was then transferred on top of the graphene spacer, via a polymethyl-methacrylate-based transfer method. Then, this mica–graphene spacer stack was lifted and transferred onto a freshly exfoliated hBN layer. This whole stack was then transferred onto a glass coverslip for imaging. The slit dimensions of the final device are shown in Fig. 5a, with a width of approximately 150 nm, length of 20 μm and height equivalent to the thickness of the graphene spacer. Supplementary Fig. 15 provide details of the fabrication flowchart and materials.

### Chemicals used

All the chemicals were purchased with the maximum purity grade available, and some were purchased in their anhydrous forms. Supplementary Table 1 provides the full list. No effect of solvent purity or residual water traces on hBN fluorescence activation was observed.

### Optical microscopy

Wide-field imaging was performed on a custom wide-field fluorescence microscope, as described elsewhere^28^. Briefly, the emitters are excited using a 561 nm laser (monolithic laser combiner 400B, Agilent Technologies), which is collimated and focused on the back focal plane of a high-numerical-aperture oil-immersion microscope objective (Olympus TIRFM 100× with a numerical aperture of 1.45). This configuration leads to wide-field illumination of the sample in a circle with ~25 μm diameter. Fluorescence emission from the sample is collected by the same objective and spectrally separated from the excitation light using dichroic and emission filters (ZT488/561rpc-UF1 and ZET488/561m, Chroma) before being projected on an electron-multiplying charge-coupled device camera (Andor iXon Ultra 897) with an electron multiplication gain of 150. An additional spectral path, mounted in parallel to the localization path, allows for the simultaneous measurements of the emission spectra from individual emitters (as described below). The sample itself is mounted in a sealed fluidic chamber, which is placed on a piezoelectric scanner (Nano-Drive, MadCityLabs) for a fine focus. The typical exposure time is 20–50 ms and the typical laser power is 10–100 mW for the wide-field excitation area of 2 × 10^3^ μm^2^, resulting in a power density of 0.35–3.50 kW cm^–2^. Unless mentioned otherwise, the illumination power density was set to 3.5 kW cm^–2^. A typical acquired image stack contained 2,000–10,000 frames.

Confocal microscopy measurements were performed in an inverted microscope configuration allowing to image samples in a liquid in custom-made chambers made of a glass ring glued onto the glass coverslip using an epoxy resin (Araldite). The excitation laser was a 561 nm picosecond diode laser (Sepia II, PicoQuant) used either in the pulsed or continuous-wave mode. The emission, after collection with a water-immersion microscope objective (Nikon SR Plan Apo 60× with numerical aperture of 1.27) was split between two fibre-coupled avalanche photodiodes (SPCM-AQRH, Excelitas) in a Hanbury Brown and Twiss configuration. Photon correlation measurements were performed using the PicoHarp time-correlated single-photon counting module (PicoQuant).

### sSMLM procedure

The image stacks acquired from the wide-field microscope were processed using ThunderSTORM^41^. The localization uncertainty of emitters is given by ${\sigma }_{{{\mbox{loc}}}} \approx {\sigma }_{{{\mbox{PSF}}}}/\sqrt{{N}_{{{\mbox{loc}}}}}$, where *σ*_PSF_ represents the radial extent of the microscope point spread function (approximately 110 nm), and *N*_loc_ is the number of photons counted in the diffraction-limited spot. Supplementary Table 2 lists all the symbols used here. In our imaging conditions, localization uncertainties ranged from 10 to 20 nm. Localizations used for emitter counting, super-resolution image rendering and trajectory analysis were filtered to keep only the localization events with 30 < *σ*_PSF_ < 200 nm and filter out artifacts. To generate super-resolved images, individual single-molecule localization microscopy localization events were rendered as 2D Gaussians with a standard deviation of 20 nm unless specified otherwise, reflecting the typical uncertainty on the emitter’s position.

sSMLM was performed following a procedure described in our previous work^28^, which we summarize here. Emitters in the spatial channel are localized using the ImageJ plugin ThunderSTORM. Briefly, a wavelet filter is applied to each frame, and peaks are then fitted by 2D-integrated Gaussians. The obtained localizations (*x*_loc_, *y*_loc_) are matched to a reference spectral point (wavelength of 650 nm) through a matrix transformation of the form (*x*_spec_, *y*_spec_) = *A* × (*x*_loc_, *y*_loc_) + *B*. Here *A* is a 2 × 2 matrix and *B* is a vector. The spectrum is then extracted as the vertical profile around (*x*_spec_, *y*_spec_) in a box of 6 × 40 pixels centred at the emitter. The pixel values are translated as spectral intensities through the linear relationship Δ*y*_spec_ ≈ *a* × *λ*, with *a* ≈ 0.25 pixels nm^–1^. This value and transformation matrix coefficients were obtained using fiducial markers emitting at known wavelengths. As described in our previous work, this was achieved by using broadband emitters (fluorescent beads) and narrow bandpass filters in the emission path. The signal-to-noise ratio of the single-emitter spectrum is evaluated and all the spectra passing a threshold are averaged to yield an ensemble spectrum. We typically used 3,000 frames for each solvent and set the threshold to *I*_loc_ > 300 photons in all the measurements.

Ensemble sSMLM spectra were maximum normalized and fit using the Python 3.7 package LMFIT^42^, to a model composed of two Lorentzians corresponding to the ZPL and PSB and a linear background. As shown in Fig. 5g,h, only the part of the normalized signal emerging more than 20% over the background was fit, as the background-induced spectra tails could pose fitting issues. For spectra in nanoslits, a spatial filter was applied to avoid counting the signal from contamination between nanoslits (Supplementary Fig. 18). The error bars on the ZPL and PSB wavelengths (Fig. 3c) as well as ZPL–PSB detuning (Supplementary Fig. 12) were obtained by evaluating the standard deviation of the parameters obtained from fitting the average of randomly chosen groups of 100 single-emitter sSMLM spectra. This ensured a sufficient signal-to-noise ratio for fitting and reflecting variations within the ensemble spectra.

### Trajectory analysis

Wide-field fluorescence frames acquired with the electron-multiplying charge-coupled device camera were first localized using ThunderSTORM^41^. Trajectories were obtained by applying the Crocker–Grier linking algorithm^43^ to the localization microscopy tables. We used the implementation of the algorithm provided by the Python package trackpy^44^. Briefly, for each localization event at a given frame, the algorithm links another event if it is found at the next frame within a specified search range. This search range was set to 120 nm in Fig. 2, as the probability of having a one-dimensional displacement exceeding this value is less than 1% according to the analysis shown in Fig. 2e.

In the statistical analysis of trajectories, the emitter position is described as a random variable of time *X*(*t*). The trajectory residence time on the crystal surface ${T}_{\,{{\mbox{res}}}\,}^{\rm{T}}$ corresponds to the duration of this single trajectory. The one-dimensional displacement probability distribution function is given by the PDF (*x*,*τ*) = *P*(*X*(*t* + *τ*) – *X*(*t*) = *x*). The residence times of the molecules at single-defect sites ${T}_{\,{{\mbox{res}}}\,}^{\rm{D}}$ were obtained using the same linking algorithm with a linking range of 35 nm (about twice the typical localization uncertainty) instead of 120 nm.

## Online content

Any methods, additional references, Nature Portfolio reporting summaries, source data, extended data, supplementary information, acknowledgements, peer review information; details of author contributions and competing interests; and statements of data and code availability are available at 10.1038/s41563-023-01658-2.

### Supplementary information

Supplementary Information Supplementary Figs. 1–18, Tables 1 and 2, captions for Supplementary Videos 1 and 2, Methods, Discussion and References.

Supplementary Video 1 Wide-field video of the crystal presented in Fig. 1 immersed in ethanol, corresponding to the data presented in Fig. 1b–d. The crystal is initially in the dark, and the continuous 3.5 kW cm^–2^ illumination is turned on at the beginning of the video, inducing a decrease in the density of emitters (Fig. 1d). The original images were acquired with 10 ms exposure time, but here we combined frames to present a lighter video with a higher signal-to-noise ratio, at the expense of a slower sampling rate (50 ms). Scale bar, 2 µm.

Supplementary Video 2 Representative wide-field video of another hBN crystal in isopropanol, taken from the steady state under 3.5 kW cm^–2^ illumination. This dataset was used for Fig. 2. The exposure time was initially set to 6 ms, but the frames were combined to obtain a similar sampling rate as above and a lighter video. Scale bar, 2 µm.

Supplementary Data 1 Data used to generate all the plots in the Supplementary Information.

### Source data

Source Data Fig. 1 Data used to generate the plots in Fig. 1.

Source Data Fig. 2 Data used to generate the plots in Fig. 2.

Source Data Fig. 3 Data used to generate the plots in Fig. 3.

Source Data Fig. 4 Data used to generate the plots in Fig. 4.

Source Data Fig. 5 Data used to generate the plots in Fig. 5.

## Data Availability

The linked localization table corresponding to Supplementary Video 2 and Fig. 2 and the corresponding raw frames are available via Zenodo at 10.5281/zenodo.8087398. Source data are provided with this paper.
