# Supplementary material for: Liquid-activated quantum emission from pristine hexagonal boron nitride for nanofluidic sensing
Source: Nat Mater. 2023 Aug 31;22(10):1236–42. doi: 10.1038/s41563-023-01658-2 (PMC10533396; doi:10.1038/s41563-023-01658-2)
Supplement: Supplementary file 1 — Supplementary Figs. 1–18, Tables 1 and 2, captions for Supplementary Videos 1 and 2, Methods, Discussion and References. [file 41563_2023_1658_MOESM1_ESM.pdf]

nature materials

Article

https://doi.org/10.1038/s41563-023-01658-2

Liquid-activated quantum emission from pristine hexagonal boron nitride for nanofluidic sensing

In the format provided by the authors and unedited

Contents

Supplementary Videos Legends **2**

Supplementary Methods **2**

Macroscopic flow response of liquid-activated hBN emitters 2

Nanoslit fabrication 2

Supplementary Discussion **3**

Recovery of the fluorescence 3

Proposed activation/photobleaching mechanism 3

Absence of photoinduced damage 3

Vibrational analysis of the emitters 4

Supplementary Figures **5**

Supplementary Figure 1. Spatial mapping of emitters in ethanol 5

Supplementary Figure 2: Characterizing the crystal surface integrity with atomic force microscopy 5

Supplementary Figure 3: Characterizing the crystal bulk integrity with Raman spectroscopy 6

Supplementary Figure 4: Fluorescence recovery in the dark 6

Supplementary Figure 5: Recovery in the dark: stability of the crystal fluorescence 7

Supplementary Figure 6: Emitter quenching by the addition of water to ethanol 8

Supplementary Figure 7: Solvent comparison through SMLM emitter counting 9

Supplementary Figure 8: Liquid activation in various organic solvents 9

Supplementary Figure 9: Solvent and light intensity dependency of the emitter density 10

Supplementary Figure 10: Macroscopic flow response of liquid-activated hBN fluorescence 11

Supplementary Figure 11: Long-lasting emitters & polarization 11

Supplementary Figure 12: Vibrational analysis of the emitters 13

Supplementary Figure 13: Affinity of emitters with dipolar molecules 13

Supplementary Figure 14: Liquid dependency of the Jablonski diagram 14

Supplementary Figure 15: Nanoslit fabrication 14

Supplementary Figure 16: Comparison of bare, masked and slit-embedded hBN emitters 14

Supplementary Figure 17: Effect of confinement on residence times 15

Supplementary Figure 18: Spatial filtering procedure for masked hBN and nanoslits 15

Supplementary Tables **17**

Supplementary Table 1: Chemicals used 17

Supplementary Table 2: List of symbols 17

Supplementary References **19**

Supplementary Videos Legend

Supplementary Video 1

Wide-field movie of the crystal presented in Figure 1 immersed in ethanol, corresponding to the data presented in Figure 1b-d. The crystal is initially in the dark, and the continuous 3.5 kW/cm^2 illumination is turned on at the beginning of the movie, inducing a decrease in the density of emitters as quantified in Figure 1d. The original images were acquired with 10 ms exposure time, but here we combined frames to present a lighter movie with a higher signal to noise ratio, at the expense of a slower sampling rate (50 ms). Scale bar: 2 microns.

Supplementary Video 2

Representative wide-field movie of another hBN crystal in isopropanol, taken from the steady state under 3.5 kW/cm^2 illumination. This data set was used for Figure 2. The exposure time was initially set to 6 ms but frames were combined to obtain a similar sampling rate as above and a lighter movie. Scale bar: 2 microns. The full raw data is available on Zenodo at https://doi.org/10.5281/zenodo.8087398.

Supplementary Methods

Macroscopic flow response of liquid-activated hBN emitters

We monitored the fluorescence of hBN crystals while driving liquid flow over their surface. For this purpose, we fabricated a microfluidic flow cell onto a coverslip, allowing to perform wide-field fluorescence imaging of a crystal encapsulated in a microchannel in which ethanol was flushed using a syringe pump (PHD Ultra, Harvard Apparatus). The flow cell was custom designed using Secure-Seal silicon imaging spacers (Grace Bio-Labs) with a thickness of H=120 µm, in which a ≈1 mm opening was made to define the channel. The spacers were sandwiched between two coverslips, and inlet and outlet tubings were connected using epoxy resin (Araldite). A sketch of the flow cell is presented in Supplementary Figure 10a. The top coverslip was omitted for clarity. By setting flow rates in the range of tens to hundreds of microliters per minute, we were able to induce laminar flows in the range of centimeters per second over the crystal, as illustrated in Supplementary Figure 10b. The surface flow rate, defined by molecular slippage, is considerably reduced by a factor of approximately $\ell_S/H \approx 10^{-4}$ where ℓ_S is the slip length of ethanol on hBN, which can be expected to be just a few nanometers[1,2]. As shown in Supplementary Figure 10c-d, the number of emitters was found to undergo a considerable increase when the crystal was submitted to 100 µL/min flow rate. Both super-resolved images were obtained by processing 1000 frames, under 1.5 kW/cm^2 illumination. The frame-wise localization microscopy counting of emitters on the crystal under flow conditions is presented in the bottom panel of Supplementary Figure 10e. The top panel shows the syringe pump flow protocol. We observed that, after a steep increase when submitted to flow, the crystal fluorescence returns to its steady state level when the liquid goes back to rest.

Nanoslit fabrication

Nanoslit devices are van der Waals (vdW) heterostructures composed of 3 layered two-dimensional crystals. All the 2D materials here are obtained by mechanical exfoliation, using adhesive tapes. The middle graphene spacer crystal is sandwiched between the top mica crystal (≈200 nm thick) and the bottom hBN crystal (≈20 nm thick) via vdW assembly. First, using e-beam lithography (EBL) and oxygen plasma etching, a few-layer graphene crystal is patterned into parallel strips with a separation of 150 nm. We then transfer the mica crystal on top of the graphene spacer, using a PMMA wet transfer method, as shown in Supplementary Figure 15 (step **a**). When choosing the top layer, it is desirable to use reasonably rectangular shaped mica crystals so as to open the slit entry on either side of the top (depicted in the optical image in **a'**). The top mica layer thickness is in the range of 150-250 nm; as a mica layer thicker than this would be less adhesive to the spacer layer, whereas a thinner layer could sag into the slits. Next, we transfer the stack of mica-graphene layer onto a freshly exfoliated bottom hBN crystal shown in Step **b**. A large crystal size of hBN is chosen as a bottom layer, so that it allows to compare emitters in the confined 2D slit (masked by spacer and top layer) with the one masked by graphene spacer only. The thickness range of the bottom hBN crystal is chosen to be between 15 nm to 25 nm. **b'** shows an optical image of the nanoslits where the bottom wall is the hBN surface, and the top slit wall is the mica surface. The slit height is determined precisely by the thickness of graphene spacer layer. In this work, we fabricated nanoslits with heights of ≈1.4 nm and ≈2.4 nm, corresponding to the four and seven graphene layers, respectively. From the AFM micrograph of the graphene spacer, the slit width can be seen as ≈150 nm and slit height ≈2.4 nm. In the final step (step **c**), we transfer the three-layer stack of top mica/graphene spacer/bottom hBN onto a glass coverslip via the PMMA transfer method, for subsequent imaging by sSMLM. The glass coverslips have pre-patterned markers of gold/chromium (thickness ≈50 nm) made by photolithography and physical deposition.

Supplementary Discussion

Recovery of the fluorescence

In order to verify that the gradual decrease in number of emitters under constant illumination reported in Figure 1d does not correspond to a degradation of the hBN crystals, we verified that the crystal fluorescence recovers when the illumination stops. For this, we recorded wide-field images and alternated 3.5 kW/cm^2 illumination periods of 20 s with variable dark times ranging from 20 s to 1 hour, and counted the fluorescence intensity per EMCCD pixel (Supplementary Fig. 4a). The inset of Supplementary Figure 4a shows the illumination protocol. We found that *(i)* the steady state remains at a constant level and *(ii)* the transient state becomes brighter with increasing dark time. As the steady state mean intensity per localization event in such conditions is 200 photons (Supplementary Fig. 4b), assuming that the fluorescence comes from emitters which contribute 200 photons per frame each, we could estimate the number of active defects per pixel by dividing the crystal intensity by this single-defect reference (Supplementary Fig. 4c). We observed that this increase in crystal fluorescence scales sub-linearly with time (power law with exponent $\approx$0.8, reaching complete recovery in about an hour), yielding a saturation around 200 active defects per square micron. This yields an upper bound for a typical inter-defect distance of $\approx$ 70 nm, which is consistent with native defect densities measured by scanning tunneling microscopy in similar pristine crystals[3]. In Supplementary Figure 5, we further verify that the steady-state density of emitters was stable during this crystal recovery in the dark (at illumination times >100s). Indeed, regardless of the dark time, upon exposure to 3.5 kW/cm^2 illumination, the crystal fluorescence reaches a similar steady state level within 10-100s. This data is consistent with the mechanism described below, where the total defect number does not change, and where the dark time dependency comes from the slow activation of emitters.

Proposed activation/photobleaching mechanism

We attribute the fluorescence recovery after photobleaching to a slow chemisorption of liquid molecules (M) onto defects (D), resulting in activated defects (MD). An activated defect in its ground state (MD) can absorb a photon with frequency ν_{exc} to reach the excited state MD*. It rapidly decays back to the ground state while emitting a photon with frequency ν_{em} and can perform this cycle millions of times as shown by photon count rates in Figure 4a. However, there is a probability that the emitter undergoes an intersystem crossing to the triplet state MD^T, which is long-lived[4] and leads to the emitter extinction upon breaking of the covalent bond between the defect and the molecule. These last two steps lead to photobleaching, and the defect returns to its optically inactive state (D), while releasing a charged molecule (M^C) to the solvent. This charged molecule undergoes surface diffusion and has a significant probability to bind to a neighboring defect. This phenomenology implies that the charged molecule M^C has a much greater reactivity with a defect D than an uncharged molecule M.

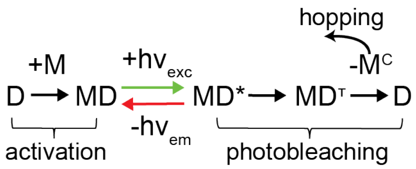

Absence of photoinduced damage

We performed a characterization of crystals deliberately exposed to conditions harsher than those in this study (20 kW/cm^2 instead of 3.5 kW/cm^2). It seems likely that hypothetical light-induced damage, like the emission, should be located at the surface. We thus performed atomic force microscopy scans of a crystal which was partially exposed to intense laser light. The light intensity modulation was performed with a reflective spatial light modulator (HOLOEYE PLUTO-2) placed on the excitation path, to obtain a checkerboard pattern as shown below in Supplementary Figure 2, where the image in (b) corresponds to localizations following the laser light exposure. The (white light) optical comparison of regions that were exposed vs. unexposed to laser light revealed no signs of light-induced damage (c). As imaged with atomic force microscopy, exposed and unexposed parts of the crystal were found to be identical in the height map (d,e), and more importantly, in the phase map of the crystal (f). AFM phase imaging did not reveal any difference between exposed and unexposed hBN, which is a qualitative indication of material uniformity[5,6]. For comparison, we could resolve the 3° phase change between glass and hBN (g). To check whether the overall crystallinity of the material could be affected in its bulk by the light, we verified through Raman spectroscopy of a thin (<10 nm) hBN crystal that the high-quality crystallinity was preserved even after 40 minutes of 20 kW/cm^2 illumination at 561 nm in ethanol. As shown in Supplementary Figure 3, the full-width at half maximum (FWHM) of the B-N stretch mode was found to be 8.3±0.4 cm^{-1}. Raman spectroscopy was not able to distinguish exposed parts of the sample from pristine crystals, all of them showing the nominal FWHM in high-quality crystals, 8 cm^{-1}, and far from strain and disorder-induced values (12 to 20 cm^{-1})[7].

Vibrational analysis of the emitters

We extend here the analysis of the emission spectra to evidence further peculiar interactions between the excited defect and solvent molecules. As shown in Supplementary Figure 12a, the energy difference between the ZPL and the PSB, which relates to the phonon dispersion of the material, was found to depend strongly on the solvent. The ZPL-PSB energy detuning ranges from 1255 cm^{-1} for nonpolar pentane to 847 cm^{-1} for polar methanol, further hinting that the phonon emission is affected by the dipolar nature of the liquid medium and, possibly, hydrogen bonding. Classically, the phonon dispersion should peak at vibrational modes of the hBN crystal, shown as dashed lines. Around 1365 cm^{-1} is the most intense vibrational mode of hBN, B-N stretching, which is both Raman and IR-active, and around 820 cm^{-1} is the IR-active out-of-plane B-N bending mode[8–10]. The phonon side band of hBN defects PL spectra, which often arises in the 150-170 meV (1200-1400 cm^{-1}) detuning range, was attributed to B-N stretching previously[11,12]. While some variations were observed throughout the literature, to the best of our knowledge, vibrational modes below 1200 cm^{-1} were not found in purely solid-state defects. Indeed, pristine hBN does not have any vibrational mode between 900 and 1200 cm^{-1} which has been dubbed the 'phonon band gap' of hBN[13]. Various bonds could yield a phonon energy in this range: functionalization of hBN nanosheets with oxygen gives rise to IR-active modes in this range[14]. A recent theoretical study proposed that the chemisorption of carbon-bearing molecules on native hBN defects can give rise to vibrational modes precisely in the 900-1100 cm^{-1} range[15]. The formation of B-C bonds seems possible as they were found to occur around 1020 cm^{-1} in boron-doped activated carbon[16] ,and carbon atoms are a common component of all activating solvents (type I and II). The apparent lowering in phonon energy with increasing solvent polarity could be explained by the progressive redistribution of emitted phonons from B-N stretching to B-C stretching. This comes with an overall decrease of the phonon-assisted emission from 57% down to 17%, quantified in Supplementary Figure 12b as the ratio of the integrated phonon side band over the integrated spectrum (inset). Overall, the vibrational signature of defects in organic solvents are compatible with covalent bonding between chemisorbed organic molecules and defect centers, as predicted by Lvova and Anina[17]. This mechanism is similar to the activation of emitters through the covalent bonding of molecules to carbon nanotubes[18]. However, as discussed in the main text, the residence time analysis at a defect suggests that this covalent bond is formed only transiently due to a decrease in desorption energy barrier by illumination.

Supplementary Figures

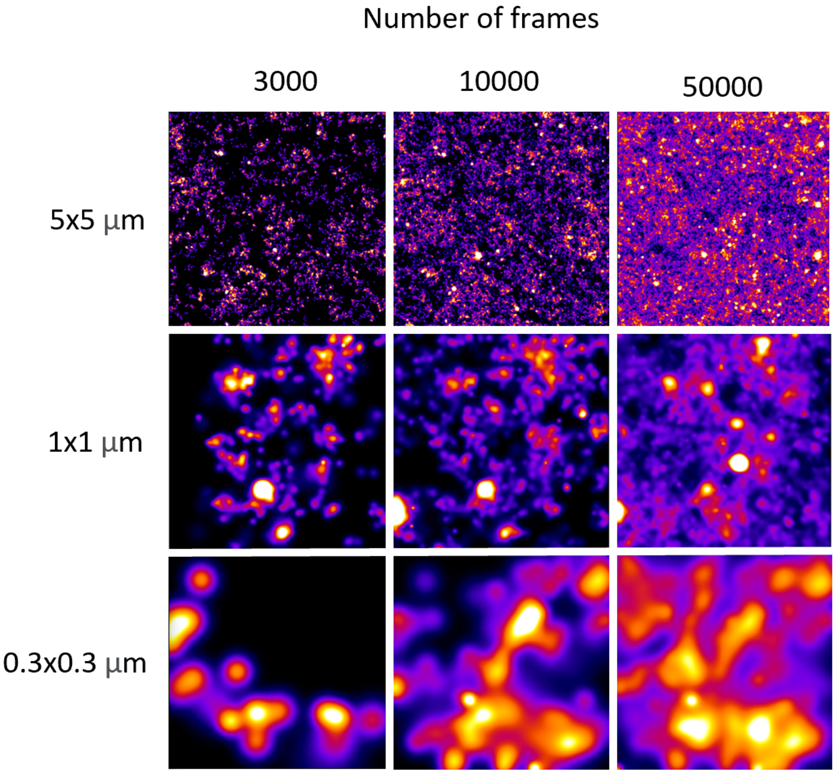

Supplementary Figure 1. Spatial mapping of emitters in ethanol. Super-resolved map of ethanol-activated emitters under 3.5 kW/cm^2 illumination for different imaging sizes ranging from 5$\times$5µm to 300$\times$300 nm and accumulation from 3k to 50k frames with 10 ms exposure. Each localization is rendered as a normalized Gaussian with a standard deviation of 15 nm to yield super-resolved probability maps used to visualize the spatial distribution of emission. We do not find a fully homogeneous defect distribution and heterogeneity is present at all scales, but there seems to be no consistent pattern appearing. As shown by the most magnified images (bottom), the dark region without localization events from the first 3000 frames eventually gets filled by localization events at later imaging times. We conclude that emitters are randomly distributed on the surface basal plane of hBN, and that the observed heterogeneities come from the long residence times at trapping sites, discussed in the main text.

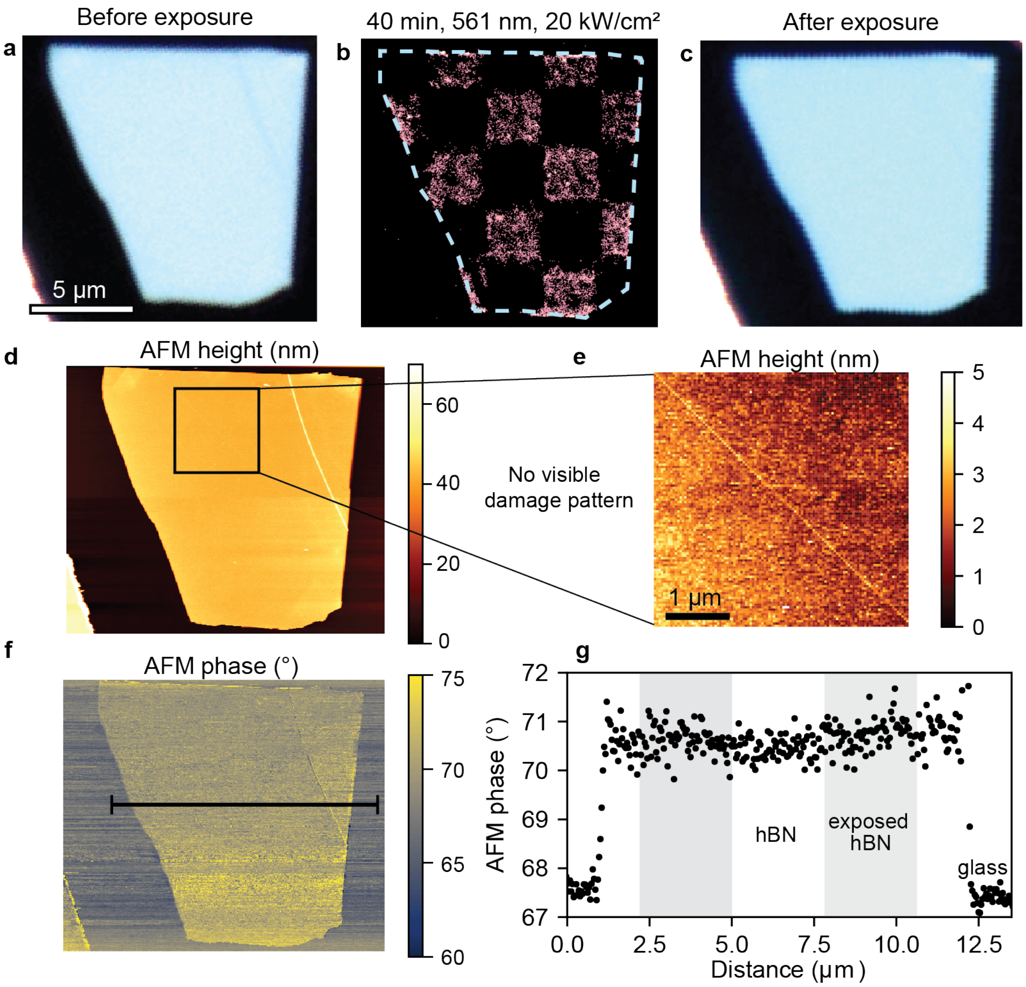

Supplementary Figure 2. AFM characterization of the integrity of the crystal surface after exposure to light in liquid. **a**, A pristine crystal was submitted to a spatially modulated 561 nm wide-field illumination of 20 kW/cm^2 for 40 minutes. Localization events from 1000 frames are presented as the super-resolved image in **b**, showing the checkerboard pattern of the spatial light modulator. No photoinduced damage was found by either optical microscopy **c** or atomic force microscopy height map **d,e** or phase map **f**. **g**, Phase profile along the line in **f**. The phase on the crystal is found to be uniform at around 70°, indicating no material change on the surface.

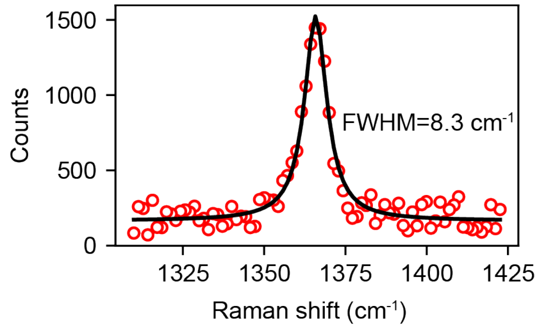

Supplementary Figure 3. Raman spectrum of hBN after intense exposure to light in ethanol. No difference is found compared with values reported for high-quality crystals in the literature[7]. The solid line is a fit to a Lorentzian curve, used to obtain the full width at half maximum of 8.3±0.4 cm^{-1}.

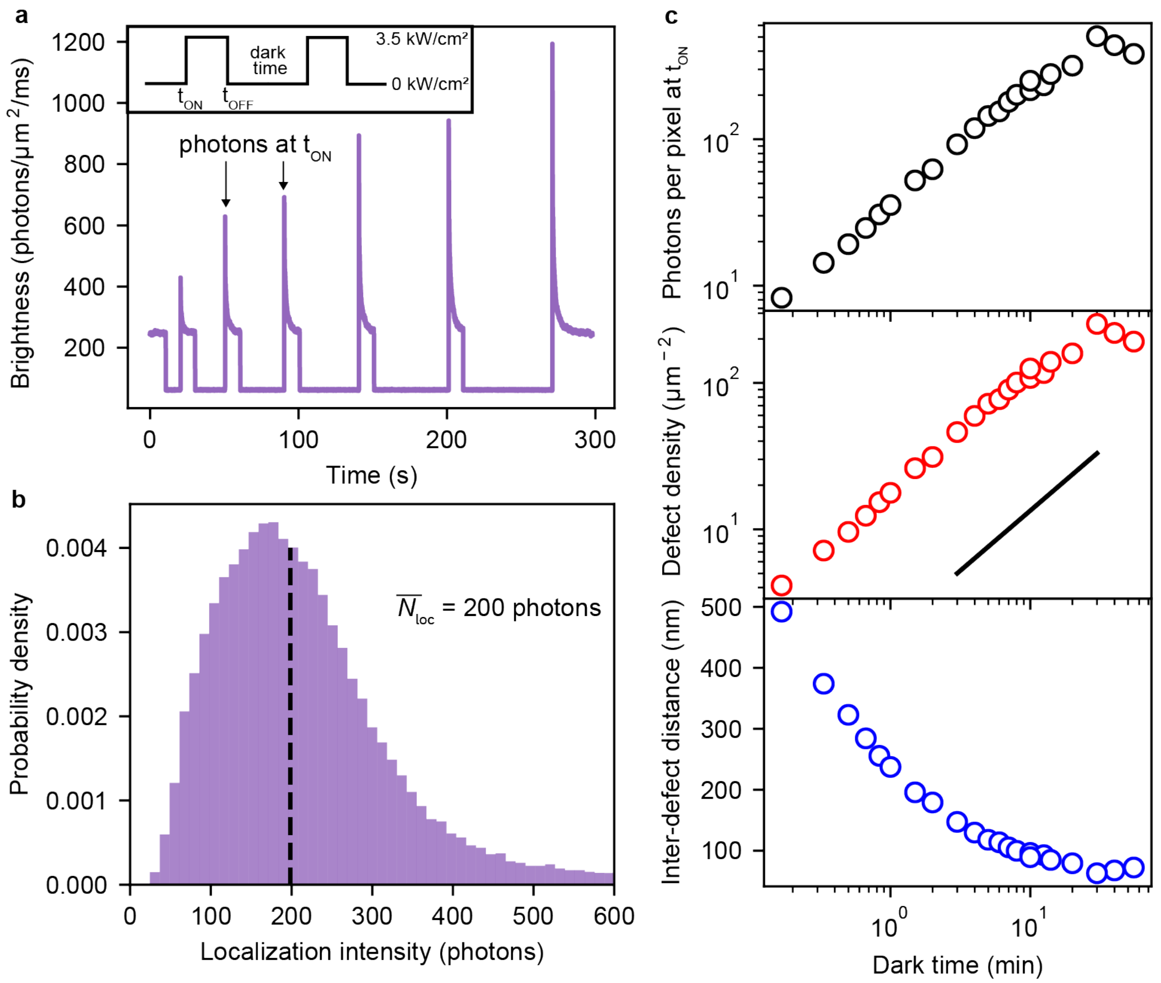

Supplementary Figure 4. Fluorescence recovery in the dark a, Counting the fluorescent average camera signal per unit area as a function of dark time, while applying the illumination protocol defined in inset. The exposure time is 6 ms. **b**, Histogram of photon counts per localization in the steady state reached after 10 seconds of illumination, as shown in Figure 1d. **c**, Top: initial brightness, appearing as spikes in **a**, as a function of dark time. Middle: this brightness can be used to roughly estimate the defect density by dividing photon counts by the mean photon count indicated by a dashed line in **b**. Bottom: average inter-defect distance obtained from **b**, assuming randomly distributed emitters.

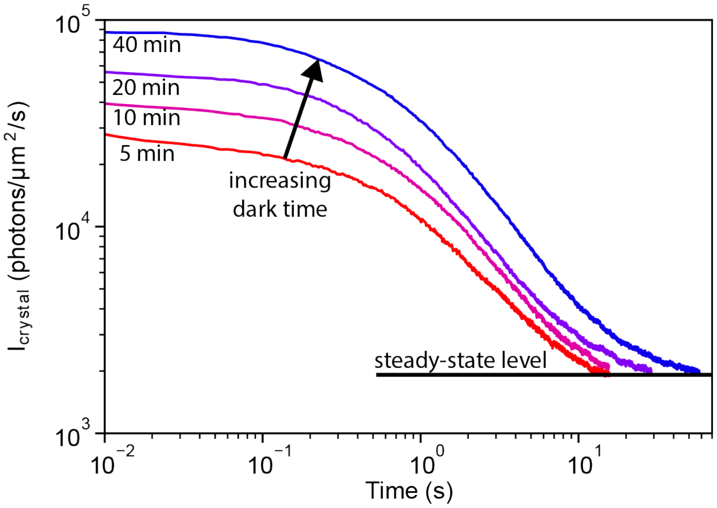

Supplementary Figure 5. Recovery in the dark: stability of the crystal fluorescence. Log-log plot of the bleaching curves corresponding to the data in Supplementary Figure 4c, showing that the dark time for recovery increases the fluorescence in the early stage of the light exposure, but leaves the steady-state unchanged.

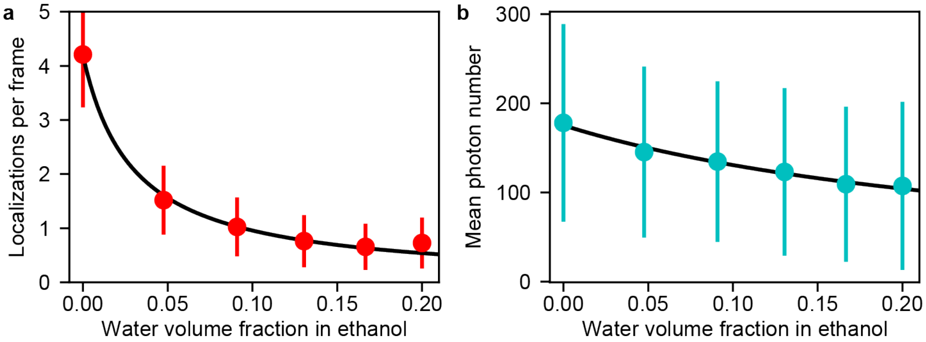

Supplementary Figure 6. hBN fluorescence quenching by addition of water to the ethanol medium. The overall fluorescence intensity drops as both **a** the localized emitter density and **b** the mean photon count of localizations drop. The experiment was conducted on a 13×13 μm region of the crystal, illuminated at 1.5 kW/cm^2. The error bars correspond to the standard deviation of the distributions over the image (space) and frames (time).

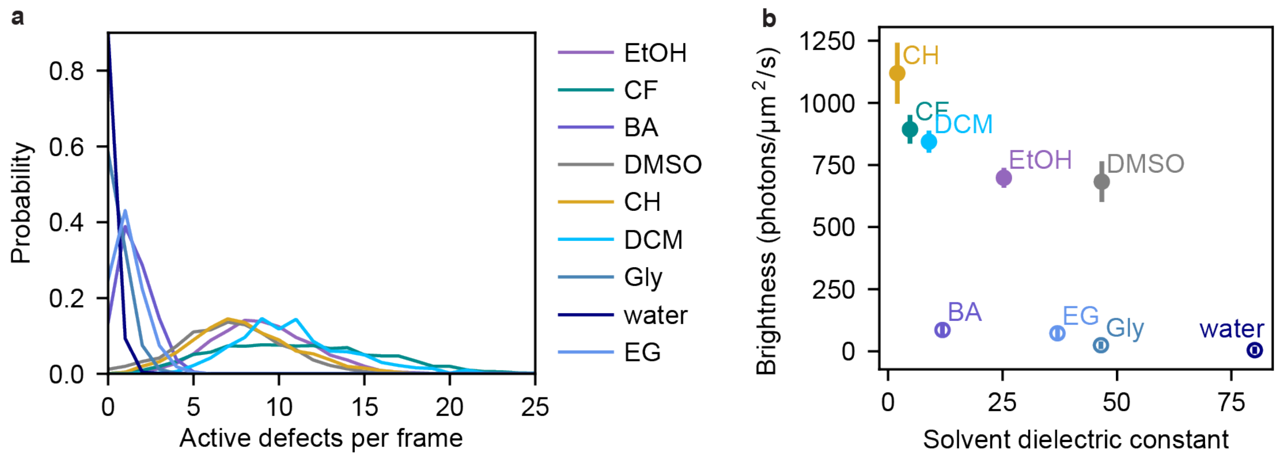

Supplementary Figure 7. Solvent comparison through SMLM emitter counting. **a**, Probability densities of the number of active emitters per 13×13 μm frame with 20 ms exposure. **b**, Crystal brightness I_{crystal} as a function of the solvent dielectric constant, showing a clear separation between type I solvents (top) and type II and III solvents (bottom). The bar chart in Figure 1e presents a crystal intensity I_{crystal} which comprises the localization intensity as well as the density of active emitters. Here the distributions of numbers of active emitters depending on the solvents are presented, clearly distinguishing type I solvents as having a high density of active emitters, unlike type II and III. Type II solvents have a non-zero average value which is better visualized in the logarithmic plot of Figure 1e. We further report a tuning of the crystal intensity with the solvent dielectric constant *among type I solvents*, which correlates with spectral changes observed in Figure 3. However, the specificity of the phenomenon, distinguishing type I solvents from types II and III, cannot be explained in terms of dielectric constant only, and exhibits some chemical specificity. Error bars correspond to standard deviations over space and time.

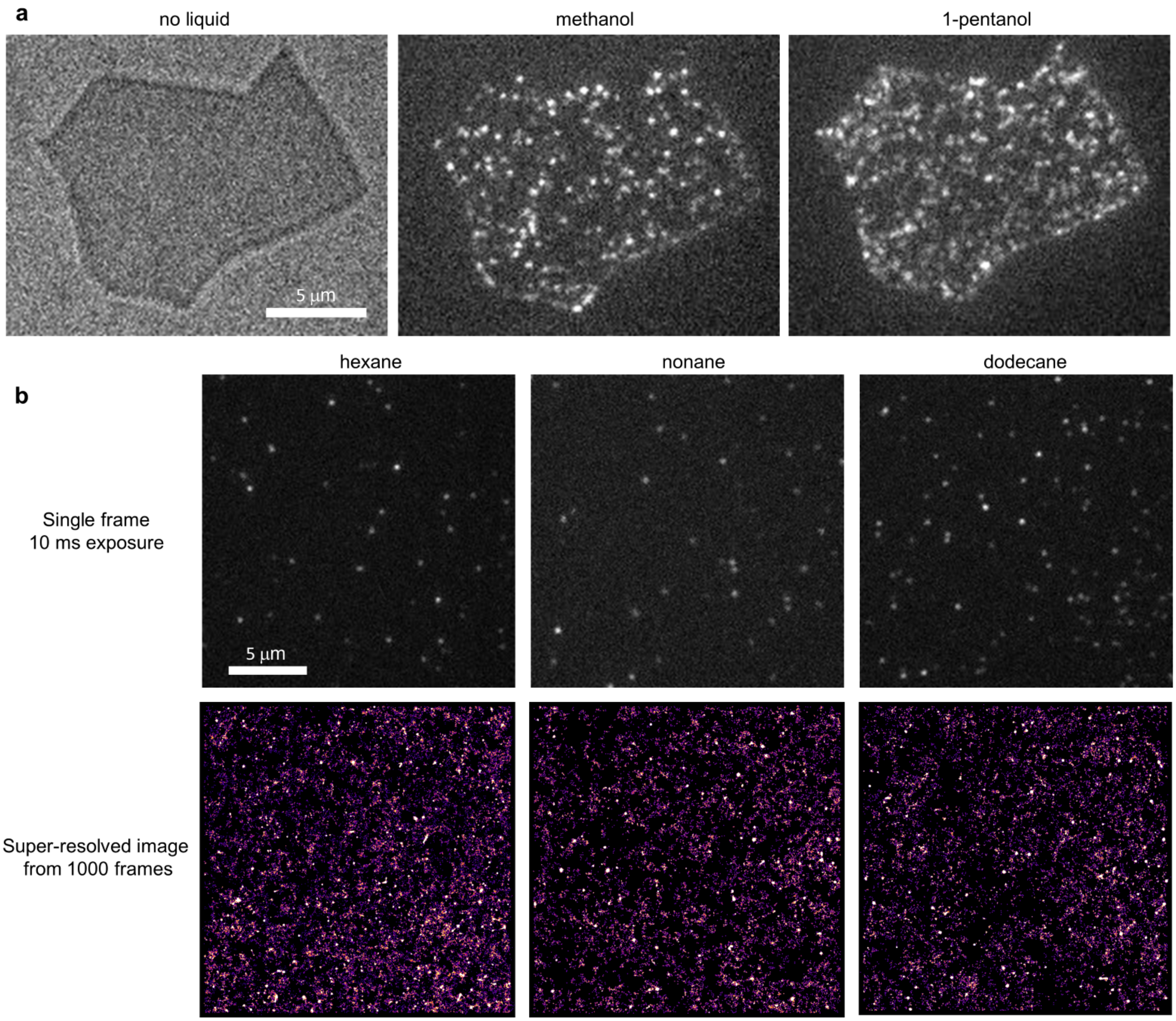

Supplementary Figure 8. Liquid activation in various organic solvents. **a**, Images of the crystal from Figure 1 in air (white light illumination), in methanol and in 1-pentanol (561 nm laser illumination). **b** Emitters were counted from 1000, 10 ms exposure frames (top) and rendered as normalized Gaussians with a standard deviation of 15 nm to yield super-resolved probability maps used to visualize the spatial distribution of emission (bottom) for hexane, nonane and dodecane, respectively. The bright spots do not coincide between liquids and seem randomly distributed as was observed for ethanol in Supplementary Figure 1. No clear effect of the chain length is found. In Figure 1e solvents are used on freshly cleaved crystals the same day under the exact same illumination conditions. Here data are measured possibly on different days and with slight alignment-induced illumination changes.

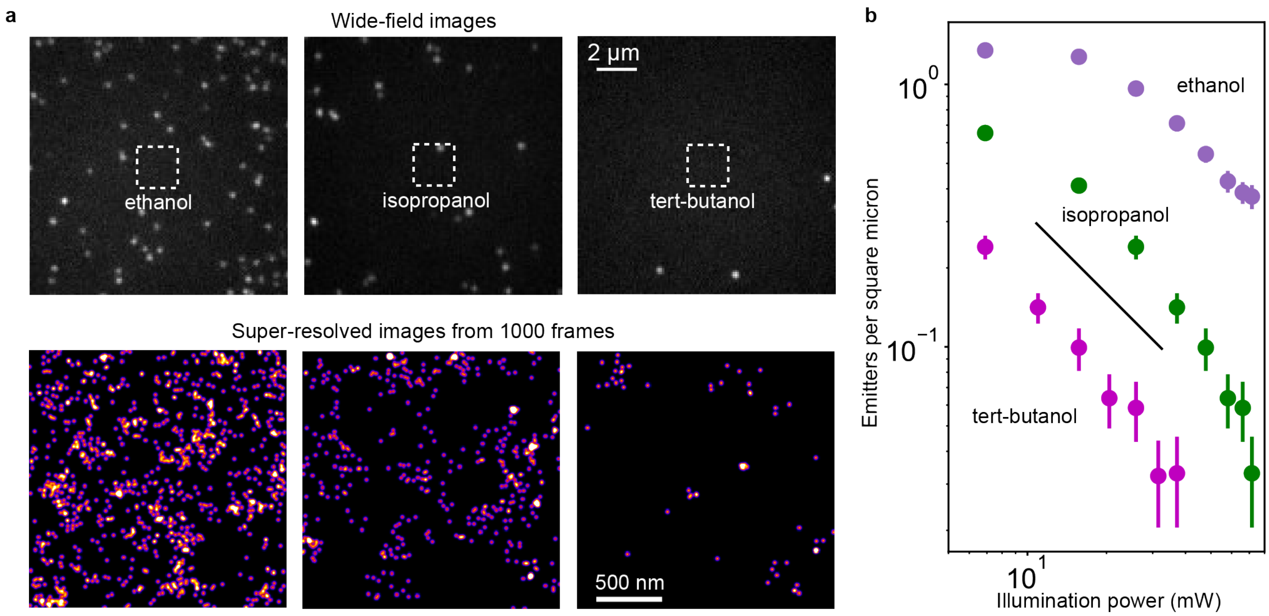

Supplementary Figure 9. Solvent and light intensity dependency of the emitter density. We observed variations in emitter density depending on *(i)* illumination and *(ii)* the solvent used. We systematically varied both parameters for a subset of three solvents: ethanol (EtOH), isopropanol (IPA) and tert-butanol (TBA). **a**, Top: 30 ms exposure frames under 3 kW/cm^2 illumination in EtOH, IPA and TBA, respectively. Bottom: super-resolved images of the region enclosed by white dashes in **a**, reconstructed from 1000 frames and rendered as normalized Gaussians with size 15 nm. A decrease in emitter density is observed for increasingly substituted alcohols. This trend contrasts with the observations on n-alkanes and primary alcohols reported above. **b**, Illumination power dependency of the emitter density for all three solvents presented on the left. The maximum power density obtained at the sample is about 3.5 kW/cm^2. The solid line indicates a slope of -1 corresponding to inverse proportionality. This light dependency is consistent with a lowered chemisorption energy at defect sites under illumination. In other words, the emitter deactivation should occur through the excited state as proposed in the mechanism in the Supplementary Discussion. Error bars correspond to the standard deviation over frames for the entire image.

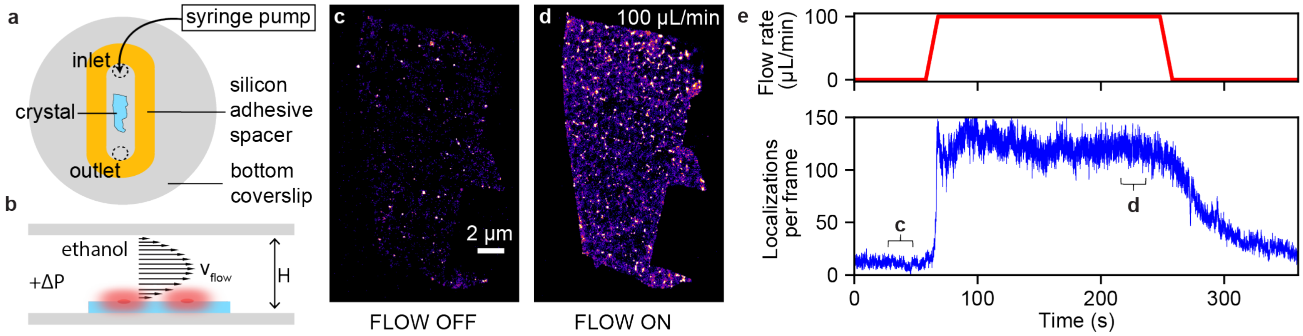

Supplementary Figure 10. Macroscopic flow response of liquid-activated hBN fluorescence. **a**, Top view sketch of the flow cell. **b** Side view sketch of the flow profile in the microchannel. **c-d**, Localization microscopy images of a hBN crystal in ethanol (c) at rest and (d) under 100 μL/min flow rate, obtained from 1000 frames. **e**, Top: flow protocol programmed with the syringe pump. Bottom: localization microscopy counting of emitters while changing flow conditions. The regions used to render (c) and (d) are indicated on the bottom graph.

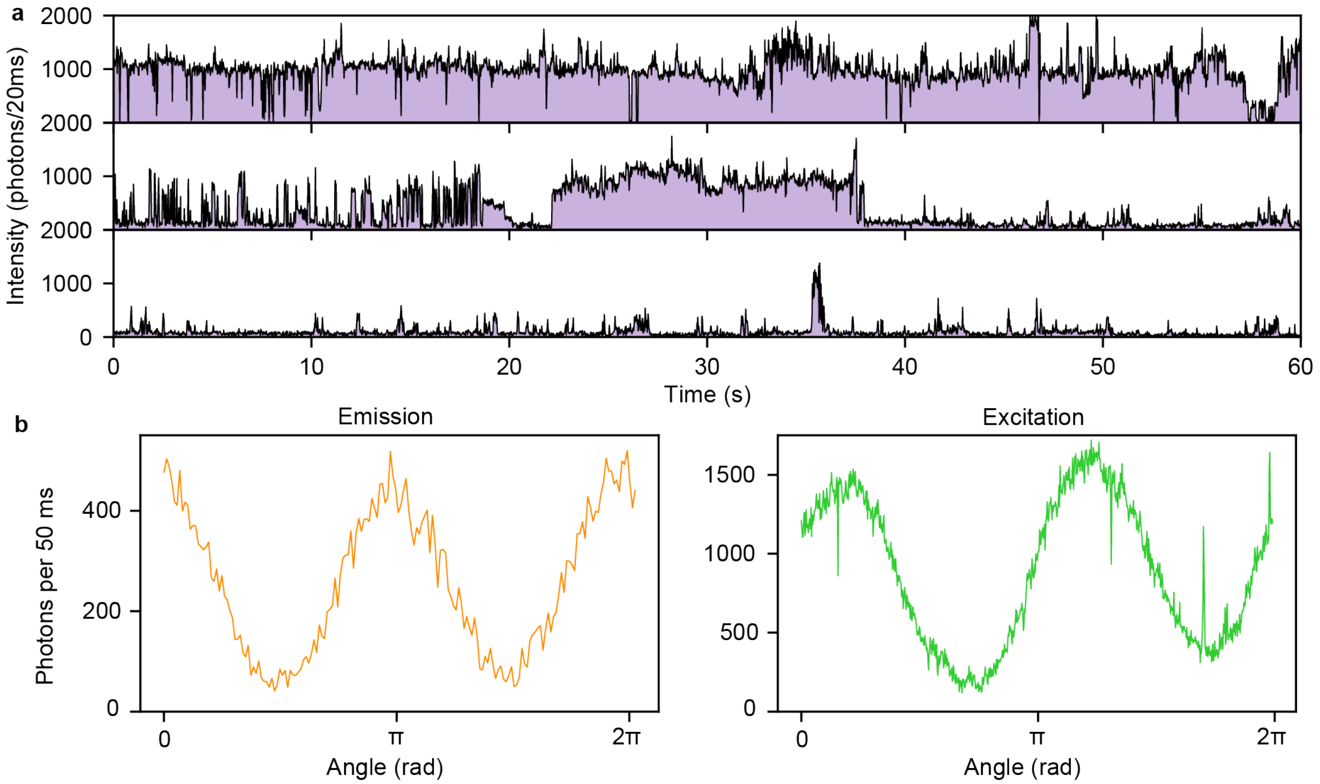

Supplementary Figure 11. Long-lasting emitters & polarization. **a**, Background-subtracted sum signal of 7x7 pixel boxes around ethanol-activated emitters under 3.5 kW/cm^2 illumination at 561 nm, showing different durations of emitter activation. While they appear as negligible in the residence times analysis as presented in Figure 2d, long-lasting traces exceeding 10 seconds were reliably found in type I solvents. **b** Using a long-lasting trace, the polarization response of emitters was measured by monitoring the fluorescent signal while rotating the polarization with a motorized half-wave plate placed before an analyzer (Thorlabs PRM1Z8, enabling rotation at 25 degrees per second). The excitation polarization response was analyzed in the same manner with the half-wave plate on the excitation path. The solvent used was pentane in both cases, and the illumination power were 0.35 and 1.4 kW/cm^2 for emission and excitation, respectively. The polar plot in Figure 3e was obtained by downsampling data to 50 angle values for clarity. Deviations from the perfect dipole fit in Figure 3e are likely due to the dichroic beamsplitter, whose birefringence was not compensated for.

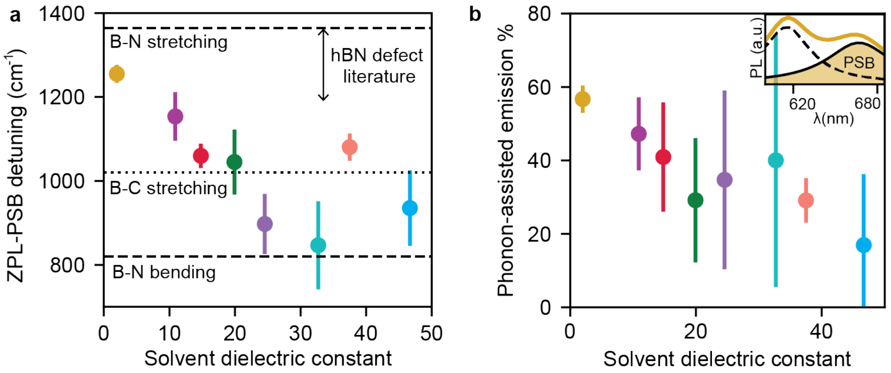

Supplementary Figure 12. Vibrational analysis of the emitters. **a**, ZPL-PSB detuning as a function of the liquid dielectric constant, reporting on the phonon dispersion around emitters. Dashed lines correspond to the Raman modes of hBN present in this energy range while the dotted line corresponds to B-C bonds as possible candidates for the observed vibrations. **b**, Quantifying the relative magnitude of phonon-assisted emission with respect to direct emission as a function of the solvent dielectric constant. The ratio is estimated as the area of the integrated PSB divided by the full integrated spectrum, as shown in the inset. Error bars correspond to the standard deviation of fitting parameters obtained by fitting averages of 100 single-molecule spectra.

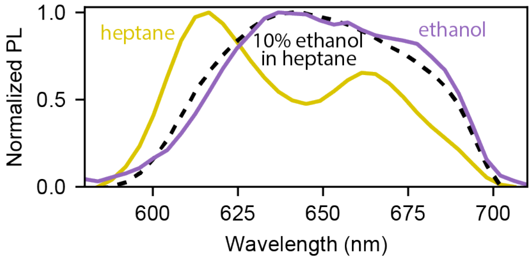

Supplementary Figure 13. Affinity of emitters with dipolar molecules. Comparison of the sSMLM spectra of heptane, ethanol and their 90:10 volume mixture. The resulting spectrum is very similar to that of ethanol, which is the minority species of the mixture. This result suggests that the charged defects possess a strong affinity towards polar solvents, leading to the enrichment in ethanol molecules within the emitter shell (diameter $\ell_{dip} \approx 1$ nm). This phenomenon, known in the dye literature as preferential solvation[19], should allow sensing of polar molecules in apolar environments.

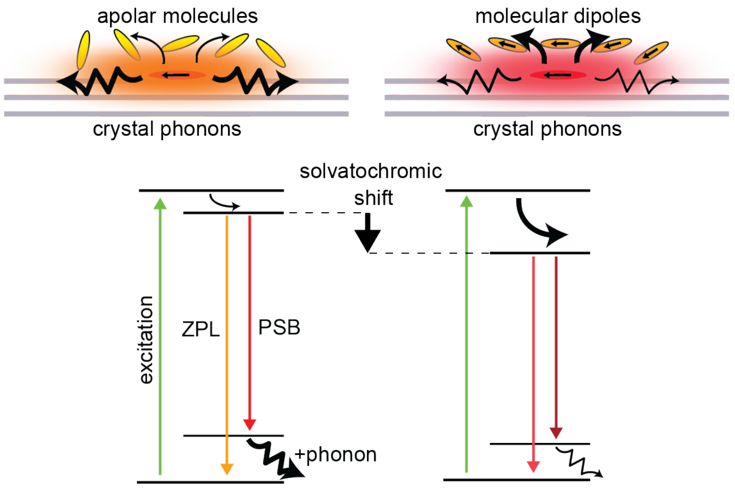

Supplementary Figure 14. Liquid dependency of the Jablonski diagram. Left: in an apolar environment such as a pure alkane, the emitter dipole is not stabilized through dipole-dipole interactions, leading to a ZPL around 615 nm (Fig. 3). Right: in a polar environment such as acetonitrile, the emitter dipole is stabilized through dipole-dipole interactions leading to a reduction in the ZPL energy, which is observed as a spectral redshift to 640 nm. As shown in Figure 3h, in apolar solvents the emitter is more strongly coupled to crystal phonons (bold zigzag arrow), whereas in polar environments the emitter is coupled preferentially to liquid molecules (bold curved arrow). This sketch is a possible explanation for positive *solvatochromism* expected to occur when the magnitude of the excited state dipole is larger than the ground state one, which is the case for $\pi - \pi^*$ transitions[19]. Solvatochromic shifts typically follow the static dielectric constant, which remains valid up to the relevant GHz regime for solvents used here[20]

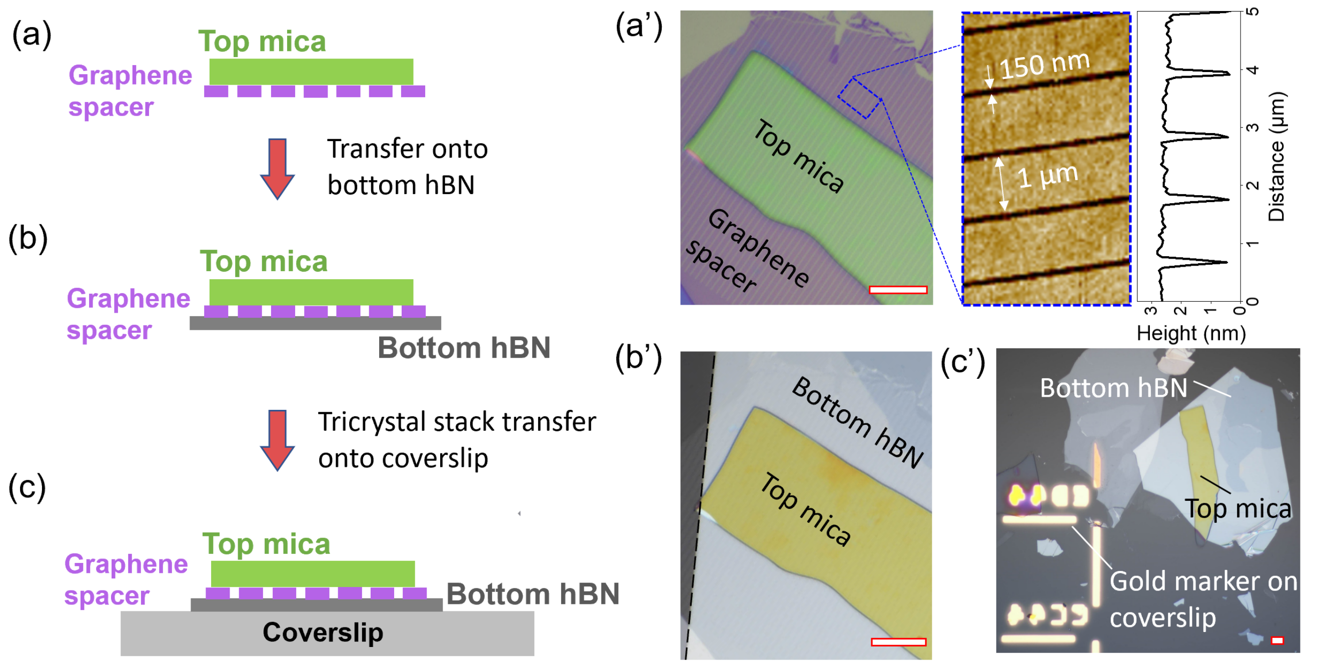

Supplementary Figure 15. Nanoslit fabrication. Step **a**: Top mica layer transferred onto pre-patterned graphene spacer (by EBL). Step **b**: Transfer of mica-graphene stack onto a freshly exfoliated bottom hBN crystal. Step **c**: Transferring the heterostructure stack onto a coverslip with pre-defined gold markers. **a'**-**c'**: Optical images of mica-graphene stack, mica-graphene-hBN stack, and mica-graphene-hBN stack on coverslip, respectively. All scale bars, 10 µm. Next to **a'**, AFM micrograph of the graphene spacer from the area marked in the blue dashed rectangle is shown along with height profile.

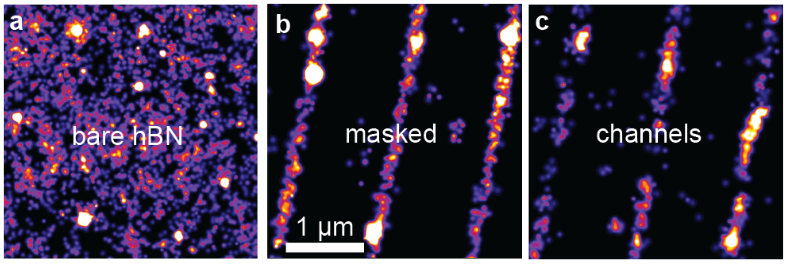

Supplementary Figure 16. Comparison of bare, masked and slit-embedded hBN emitters. **a**, Super-resolved image from 10k frames of acetonitrile-activated emitters under 0.7 kW/cm^2 illumination with 20 ms exposure time. **b**, Graphene-masked hBN imaged in the same conditions. **c**, 2.4 nm-high nanoslit imaged in the same conditions. We evidence here that the uncovered part of masked hBN truly behaves as bare hBN, as shown by the agreement of bare and masked hBN spectra in Figure 5h-i. Nanoslits do exhibit a reduced number of emitters, but these emitters are not quenched and no photons are lost (less than 1%), as shown by the histograms in Figure 5e. The heterogeneities observed here come from under-sampling of the bottom hBN surface defects due to the reduced number of active emitters, illustrated in Supplementary Figure 1.

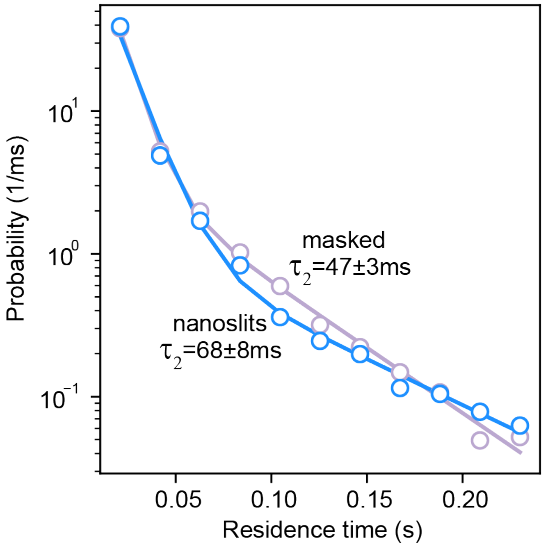

Supplementary Figure 17. Effect of confinement on residence times. Single-defect residence time T^D_{res} distribution of emitters on masked hBN (purple) and nanoslits (blue) were fit to a two-component exponential decay as in Figure 3d. Residence times were obtained by applying the tracking procedure to the geometrically filtered emitters with an uncertainty-limited cutoff distance set to 35 nm for both datasets. Residence times were found to be affected by confinement as the nanoslit-confined emitters exhibited a long exponential decay time constant of τ^D_{res} =68±8 ms while unconfined emitters on the same image had a τ^D_{res} =47±3 ms time constant. This confinement-induced increase in residence time cannot explain the observed confinement-induced decrease in number of emitters shown in Figure 5e. This means that the leading effect of confinement is the decrease in activation rate, rather than the photobleaching kinetics. This observation echoes the result of the flow measurement, where the number of active emitters was found to increase dramatically when the fluid was driven across the crystal surface, suggesting that the exchange between bulk and surface liquid molecules is affecting the number of active emitters.

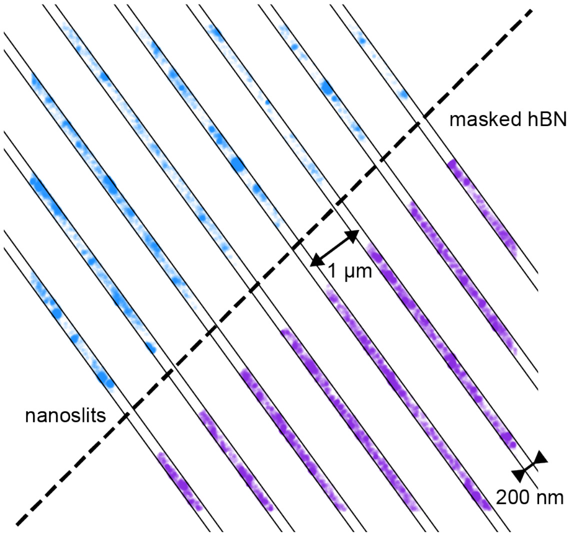

Supplementary Figure 18. Spatial filtering procedure for masked hBN and nanoslits. Super-resolved image from the edge of the nanoslits used for the quantitative comparison performed in Figure 5e, overlaid with the mask applied for counting. The emitter density in nanoslits amounts to about one third of its value on bare hBN. For emitter counting and spectral assignments in samples with defined patterns, we filtered localizations spatially, effectively applying a post-processing mask matching the graphene pattern. The filter was set to 200 nm width to account for the slit width (150 nm) and twice the localization uncertainty of about 25 nm. This ensures that we did not assign counts or spectra to localization events occurring in between nanoslits, which can be seen in Supplementary Figure16c for instance. These emitters are likely due to transfer-induced contamination, which is inevitable in fabrication but kept to low density.

Supplementary Tables

Supplementary Table 1: Chemicals used

Solvent	Supplier	hBN activation type
pentane (anhydrous)	Sigma-Aldrich	Type I
hexane	Sigma-Aldrich	Type I
heptane (anhydrous)	Sigma-Aldrich	Type I
nonane	Sigma-Aldrich	Type I
decane	Sigma-Aldrich	Type I
dodecane	Sigma-Aldrich	Type I
hexadecane (anhydrous)	Sigma-Aldrich	Type I
methanol	Sigma-Aldrich	Type I
methanol (anhydrous)	Sigma-Aldrich	Type I
ethanol	Sigma-Aldrich	Type I
ethanol (anhydrous)	Fisher Scientific	Type I
1-propanol (anhydrous)	Sigma-Aldrich	Type I
1-butanol (anhydrous)	Sigma-Aldrich	Type I
1-pentanol	Sigma-Aldrich	Type I
isopropanol	Sigma-Aldrich	Type I
tert-butanol	Sigma-Aldrich	Type I
acetone	Sigma-Aldrich	Type I
acetonitrile (anhydrous)	Sigma-Aldrich	Type I
dimethylsulfoxide	Sigma-Aldrich	Type I
dimethylformamide	Sigma-Aldrich	Type I
cyclohexane	Sigma-Aldrich	Type I
chloroform	Sigma-Aldrich	Type I
dichloromethane	Sigma-Aldrich	Type I
chloroform	Sigma-Aldrich	Type I
ethylene glycol	Sigma-Aldrich	Type II
glycerol	Sigma-Aldrich	Type II
benzyl alcohol	Sigma-Aldrich	Type II
propane-1,3-diol	Sigma-Aldrich	Type II
deionized water	MilliQ	Type III
deuterium oxide	Sigma-Aldrich	Type III
hydrogen peroxide	Sigma-Aldrich	Type III

Supplementary Table 2: List of symbols

Super-resolution

N_{loc}	Number of photons of a localization event
σ_{PSF}	Point spread function radial standard deviation
$\sigma_{\text{loc}} \approx \sigma_{\text{PSF}}/\sqrt{N_{\text{loc}}}$	Uncertainty of localization
$I_{\text{crystal}} = \sum_{\text{frame}} N_{\text{loc}}/S\Delta t$	Crystal brightness

Tracking

x	1D displacement coordinate
t	Time coordinate
τ	Lag time
$P()$	Probability notation
$PDF(x,\tau) = P\Big(X(t+\tau) - X(t) = x\Big)$	1D displacement probability density function
D	Diffusion coefficient
T_{res}^{D}	Residence time at a single defect
T_{res}^{T}	Residence time for a whole trajectory
τ_{res}^{D}	Long exponential time constant in the distribution of T_{res}^{D}
τ_{res}^{T}	Long exponential time constant in the distribution of T_{res}^{T}

Liquids - nanoslits

h	Nanoslit height
w	Nanoslit width
$\varepsilon_{\mathrm{liq}}$	Bulk static dielectric constant
$\varepsilon_{\mathrm{conf}}$	Confined static dielectric constant
$\varepsilon_{\mathrm{wall}}$	Mica top wall static dielectric constant
μ_D	Defect dipole moment
μ_S	Solvent molecule dipole moment
ℓ_{dip}	Range of dipolar interactions

Supplementary References

1. Secchi, E., Marbach, S., Niguès, A., Stein, D., Siria, A. & Bocquet, L. Massive radius-dependent flow slippage in carbon nanotubes. *Nature* **537**, 210–213 (2016).

2. Seal, A. & Govind Rajan, A. Modulating water slip using atomic-scale defects: Friction on realistic hexagonal boron nitride surfaces. *Nano Lett.* **21**, 8008–8016 (2021).

3. Wong, D. *et al.* Characterization and manipulation of individual defects in insulating hexagonal boron nitride using scanning tunnelling microscopy. *Nat. Nanotechnol. 2015 10:11* **10**, 949–953 (2015).

4. Demchenko, A. P. Photobleaching of organic fluorophores: Quantitative characterization, mechanisms, protection. *Methods Appl. Fluoresc.* **8**, 022001 (2020).

5. Magonov, S. N., Elings, V. & Whangbo, M. H. Phase imaging and stiffness in tapping-mode atomic force microscopy. *Surf. Sci.* **375**, L385–L391 (1997).

6. Pang, G. K. H., Baba-Kishi, K. Z. & Patel, A. Topographic and phase-contrast imaging in atomic force microscopy. *Ultramicroscopy* **81**, 35–40 (2000).

7. Caldwell, J. D., Aharonovich, I., Cassabois, G., Edgar, J. H., Gil, B. & Basov, D. N. Photonics with hexagonal boron nitride. *Nat. Rev. Mater.* **4**, 552–567 (2019).

8. Geick, R., Perry, C. & Rupprecht, G. Normal modes in hexagonal boron nitride. *Phys. Rev.* **146**, 543 (1966).

9. Reich, S., Ferrari, A., Arenal, R., Loiseau, A., Bello, I. & Robertson, J. Resonant raman scattering in cubic and hexagonal boron nitride. *Phys. Rev. B* **71**, 205201 (2005).

10. Serrano, J., Bosak, A., Arenal, R., Krisch, M., Watanabe, K., Taniguchi, T., Kanda, H., Rubio, A. & Wirtz, L. Vibrational properties of hexagonal boron nitride: inelastic X-ray scattering and ab initio calculations. *Phys. Rev. Lett.* **98**, 095503 (2007).

11. Chejanovsky, N. *et al.* Structural attributes and photodynamics of visible spectrum quantum emitters in hexagonal boron nitride. *Nano Lett.* **16**, 7037–7045 (2016).

12. Martínez, L., Pelini, T., Waselowski, V., Maze, J., Gil, B., Cassabois, G. & Jacques, V. Efficient single photon emission from a high-purity hexagonal boron nitride crystal. *Phys. Rev. B* **94**, 121405 (2016).

13. Vuong, T., Cassabois, G., Valvin, P., Ouerghi, A., Chassagneux, Y., Voisin, C. & Gil, B. Phonon-photon mapping in a color center in hexagonal boron nitride. *Phys. Rev. Lett.* **117**, 097402 (2016).

14. Sainsbury, T., Satti, A., May, P., Wang, Z., McGovern, I., Gun'ko, Y. K. & Coleman, J. Oxygen radical functionalization of boron nitride nanosheets. *J. Am. Chem. Soc.* **134**, 18758–18771 (2012).

15. Jiang, T., Le, D., Rawal, T. B. & Rahman, T. S. Syngas molecules as probes for defects in 2D hexagonal boron nitride: their adsorption and vibrations. *Phys. Chem. Chem. Phys.* **23**, 7988–8001 (2021).

16. Romanos, J. *et al.* Infrared study of boron–carbon chemical bonds in boron-doped activated carbon. *Carbon* **54**, 208–214 (2013).

17. Lvova, N. & Ananina, O. Y. Theoretical study of the adsorption properties of porous boron nitride nanosheets. *Comput. Mater. Sci.* **115**, 11–17 (2016).

18. Piao, Y., Meany, B., Powell, L. R., Valley, N., Kwon, H., Schatz, G. C. & Wang, Y. Brightening of carbon nanotube photoluminescence through the incorporation of sp^3 defects. *Nat. Chem.* **5**, 840–845 (2013).

19. Nigam, S. & Rutan, S. Principles and applications of solvatochromism. *Appl. Spectrosc.* **55**, 362A–370A (2001).

20. Yomogida, Y., Sato, Y., Nozaki, R., Mishina, T. & Nakahara, J. Dielectric study of normal alcohols with THz time-domain spectroscopy. *J. Mol. Liq.* **154**, 31–35 (2010).
